# Ackr3-Venus knock-in mouse lights up brain vasculature

**DOI:** 10.1186/s13041-021-00862-y

**Published:** 2021-09-28

**Authors:** Aliza T. Ehrlich, Meriem Semache, Pierre Couvineau, Stefan Wojcik, Hiroyuki Kobayashi, Marcus Thelen, Florence Gross, Mireille Hogue, Christian Le Gouill, Emmanuel Darcq, Michel Bouvier, Brigitte L. Kieffer

**Affiliations:** 1 Douglas Research Center, McGill University, Montréal, Canada; 2 Institute for Research in Immunology and Cancer (IRIC) and Department of Biochemistry and Molecular Medicine, Université de Montréal, Montréal, Québec H3T 1J4 Canada; 3 University of Surrey, Guildford, UK; 4 Present Address: Institute for Research in Biomedicine, Università della Svizzera Italiana, Bellinzona, Switzerland; 5 Present Address: Oxford Brookes University, Oxford, UK; 6 Present Address: INSERM U1114, University of Strasbourg, Strasbourg, France; 7 Present Address: Domain Therapeutics North America, Montréal, Québec H4S 1Z9 Canada; 8 Present Address: University of California, San Francisco, USA

**Keywords:** CXCR7, GPCR, Signaling, G protein, βarrestin, GABA, Endothelial cells

## Abstract

The atypical chemokine receptor 3, ACKR3, is a G protein-coupled receptor, which does not couple to G proteins but recruits βarrestins. At present, ACKR3 is considered a target for cancer and cardiovascular disorders, but less is known about the potential of ACKR3 as a target for brain disease. Further, mouse lines have been created to identify cells expressing the receptor, but there is no tool to visualize and study the receptor itself under physiological conditions. Here, we engineered a knock-in (KI) mouse expressing a functional ACKR3-Venus fusion protein to directly detect the receptor, particularly in the adult brain. In HEK-293 cells, native and fused receptors showed similar membrane expression, ligand induced trafficking and signaling profiles, indicating that the Venus fusion does not alter receptor signaling. We also found that ACKR3-Venus enables direct real-time monitoring of receptor trafficking using resonance energy transfer. In ACKR3-Venus knock-in mice, we found normal ACKR3 mRNA levels in the brain, suggesting intact gene transcription. We fully mapped receptor expression across 14 peripheral organs and 112 brain areas and found that ACKR3 is primarily localized to the vasculature in these tissues. In the periphery, receptor distribution aligns with previous reports. In the brain there is notable ACKR3 expression in endothelial vascular cells, hippocampal GABAergic interneurons and neuroblast neighboring cells. In conclusion, we have generated *Ackr3-Venus* knock-in mice with a traceable ACKR3 receptor, which will be a useful tool to the research community for interrogations about ACKR3 biology and related diseases.

**Supplementary Information:**

The online version contains supplementary material available at 10.1186/s13041-021-00862-y.

## Introduction

G protein-coupled receptors (GPCRs) constitute the largest family of cell surface receptors. They are involved in a wide diversity of cellular and physiological processes and contribute to disease. GPCRs are the target of 35% of commercially marketed drugs [1]. Since this family of proteins are an essential component of the mechanisms regulating physio-pathological processes, it is necessary to have tools allowing their study in physiologically relevant systems. The atypical chemokine receptor (ACKR3) [2] previously known as chemokine receptor type 7 (CXCR7), is a class A, rhodopsin-like GPCR that was first isolated in 1989 from dog thyroid cDNA libraries [3, 4] and originally called RDC1 when it was identified as an orphan receptor [5]. ACKR3 was deorphanized as a low-affinity receptor for the CXCR3 ligand, CXCL11, and high-affinity receptor for the ligand of CXCR4, SDF-1α/CXCL12 (for simplicity, CXCL12 henceforth) [6, 7]. CXCR3 and CXCL11 have reported roles in tumor development and maintenance and ACKR3 has been proposed to scavenge excess CXCL11 [8]. CXCR4 and ACKR3 binding to endogenous ligand CXCL12 has been shown to contribute to tumor growth, neovascularization, neurogenesis and brain inflammation [9–11]. Thus, ACKR3 ligands are considered promising drugs in the context of cancer and neuroinflammation yet mechanistic understanding of ACKR3 biology is incomplete, and the potential of ACKR3 as a therapeutic target has not been fully exploited.

Unlike many other chemokine receptors, ACKR3 lacks the ability to mediate chemotaxis and calcium mobilization after ligand binding. Akin to other atypical chemokine receptors, ACKR3 is not coupled to any G protein but can induce MAP-kinase and AKT activation through a βarrestin dependent signaling pathway [12]. ACKR3 has been reported to undergo constitutive ubiquitination which is required for membrane localization of the receptor and agonist dependent βarrestin mediated internalization and recycling [13]. It has been shown that ACKR3 forms heterodimers with CXCR4 leading to an increase in βarrestin engagement, cell migration, an inhibition of CXCR4 signaling and mediating sustained activation of ERK1/2 and p38 MAPK signaling pathways [14, 15]. Additionally, ACKR3 scavenges and degrades excess CXCL12, lowering the level of CXCR4 activity [16, 17], and pharmacological inhibition of the binding of CXCL12 to ACKR3 increases the amounts of CXCL12 in blood plasma [18, 19]. Thus, ACKR3 has three established cellular functions, it recruits βarrestin, it mediates CXCR4 signaling through heterodimer modulation and it scavenges excess CXCL12.

With considerable drug development efforts underway for anti-cancer ACKR3 therapeutics [20] and the potential to target other disorders, including brain diseases [21–26] there is the need for improved tools to study ACKR3 ex vivo and in vivo [27]. In fact, methods to study ACKR3 expression, signaling and function in physiological conditions are scarce. Conventional immunohistochemical methods are not sensitive enough to detect ACKR3 in most organs and few ACKR3 antibodies specifically detect ACKR3 in tissue [19]. The widely used mouse monoclonal ACKR3 antibody (118G) relies on signal amplification steps which can identify ACKR3 + cells but lacks receptor level detection [19]. Furthermore, mouse monoclonal antibodies can non-specifically interact with mouse IgG making interpretation of results in living or fixed mouse tissues difficult. In terms of mouse tools, genetically modified mice that are currently available are reporter mouse lines where ACKR3-positive cells, but not the receptor, are labelled with the exception of an HA-Ackr3 knock-in mouse which provided the first localization of ACKR3 in the embryonic mouse brain [28]. A *Cxcr7*^+*/lacZ*^ mouse line was reported, in which the promoter for *Cxcr7* drives lacZ expression allowing for the identification of *Cxcr7* + cells with β-galactosidase staining [19, 29]. GENSAT has created a BAC transgenic line, so-called *Cxcr7-EGFP* mice, where the EGFP is introduced upstream of the start codon of *Cxcr7* resulting in expression of EGFP under the control of the *Cxcr7* promoter and regulatory elements (The Gene Expression Nervous System Atlas project GENSAT; http://www.gensat.org/index.html) [30]. A *Cxcr7-GFP/* + knock-in mouse line was reported, in which one copy of the *Cxcr7* gene was replaced with cDNA encoding enhanced GFP leading to a heterozygous receptor knockout and EGFP expression in CXCR7-expressing cells [31]. Finally, the HA-Ackr3 and HA-Ackr3-ST/A (phosphorylation deficient) mouse lines were created to investigate the role of phosphorylation of ACKR3 in vivo [28]. The immunodetection of ACKR3 by immunostaining for HA was first evaluated in cells prior to making the animals [32], and then in HA-Ackr3 animals where the receptor protein is visible as clustering on cells in the embryonic mouse brain [28]. To our knowledge, therefore, all the genetically modified mice that are currently available can detect ACKR3 indirectly, either by expression of ACKR3-expressing cells or by ex-vivo immunodetection of the ACKR3 receptor protein in embryonic brain but an anatomical description of the receptor expression by direct observation in mature adult mouse brain is lacking.

To this end, we engineered a knock-in mouse expressing ACKR3 fused at its C-tail to a yellow fluorescent protein, Venus. These animals were created to trace ACKR3 expression in the brain and allow monitoring of receptor trafficking and signaling ex vivo and in vivo*.* Here, we characterize *Ackr3-Venus* animals and we report the first, to our knowledge, direct observation of receptor expression across the adult mouse body. In peripheral organs, we find highest receptor expression in spleen, in particular the red pulp and marginal zone B cells. In the brain, we found receptors localize to neuroblast neighboring cells in the olfactory bulb and hippocampus, GABAergic interneurons of the hippocampus and, vascular CD31 + endothelial cells. ACKR3-Venus animals allow for the direct observation of receptors in vivo without the need for immunodetection and will therefore be a unique tool to probe open questions about ACKR3 function in neuroscience, cardiovascular system, and cancer.

## Results

### ACKR3-Venus lacks G protein activity but recruits βarrestins

**Fig. 1:**
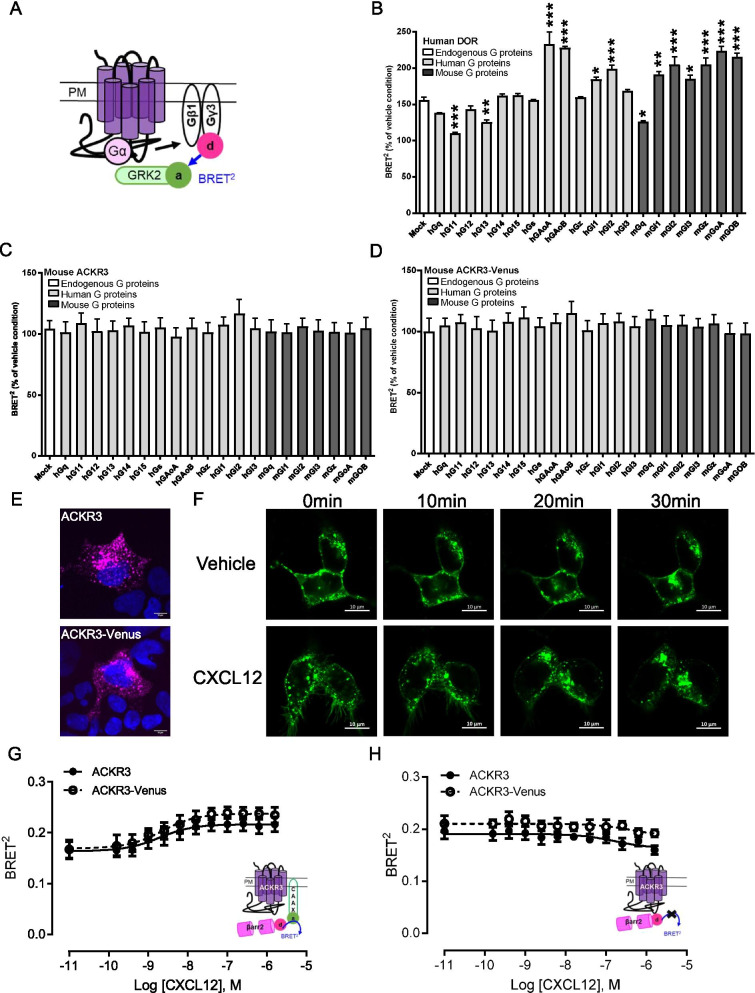
Functional characterization of ACKR3-Venus in HEK-293 cells. **A** Schematic representation of the BRET^2^ biosensor used to assess receptor induced G protein activation by monitoring the interaction between the dissociated hGγ3-RlucII (BRET^2^ sensor donor) upon activation and hGRK2-GFP10 (BRET^2^ sensor acceptor) [33, 78]. **B**–**D** HEK-293 cells were co-transfected with the indicated receptor (DOR, ACKR3 or ACKR3-venus), Gβ1, hGγ3-RlucII, hGRK2-GFP10 and the indicated human (h) or mouse (m) Gα subunits and then stimulated by agonist (see **B** and **C**, **D** below). Mock is a condition with cells not co-transfected with a Gα subunit encoding cDNA, thus detecting receptor-mediated activation of endogenous G proteins. Data are expressed as % of vehicle condition (Mean ± SEM, see Additional file 1: Figure S1A–C for vehicle conditions at each G protein), *p < 0.05, **p < 0.01, ***p < 0.0001 vs Mock condition, in 3 independent experiments. **B** G protein activation profile of the human Delta opioid receptor (DOR) following a 10 min stimulation with 30 µM of Met-Enkephalin. **C**, **D** G protein activation profile of the untagged (ACKR3) or Venus-tagged (ACKR3-Venus) mouse ACKR3 following a 10 min stimulation with 1 µM CXCL12. **E** Surface Flag ACKR3 (top) or Flag-ACKR3-Venus (bottom) receptors were labelled before fixation and permeabilization in HEK-293 cells to detect the surface pool of receptors only, using confocal microscopy. **F** HEK-293 cells were transfected with ACKR3-Venus and the time-dependent changes in the subcellular distribution of ACKR3-Venus was monitored using confocal microscopy in cells stimulated with 300 nM CXCL12 (bottom) and vehicle-treated control (top). **G**, **H** βarrestin2 recruitment assessed using the βarrestin2-RlucII/rGFP-CAAX ebBRET biosensor [34]. HEK-293 cells were transfected with either untagged or Venus-tagged mouse ACKR3 receptors, βarrestin2-RlucII, with (**G**) or without (**H**) the rGFP-CAAX BRET^2^ biosensor plasmid. Cells were stimulated for 10 min with increasing concentrations of CXCL12. Upon receptor activation, βarrestin2-RlucII (donor) is recruited to the plasma membrane (rGFP-CAAX, acceptor), resulting in an increase BRET^2^ signal. The LogEC_50_ values for the CXCL12-promoted βarrestin2 are 8.7 (CI 8.9–8.5) and 8.6 (CI 8.8–8.4); *P* = 0.96. The control experiment (**H**), with no rGFP-CAAX expression, is to rule out a potential contribution of the Venus tagged to ACKR3 to the ebBRET signal measured between βarrestin2-RlucII and rGFP-CAAX. Data are expressed as raw BRET signal (Mean ± SEM) from 3 independent experiments

To test the functionality of the ACKR3-Venus construct used to generate the KI mice, the ACKR3 receptor genetically fused to the fluorescent protein, Venus-GFP (Venus), at its C-tail was expressed in HEK-293 cells and it signaling properties compared with that of the wild-type (WT) ACKR3. For this purpose, a BRET^2^ assay [33–36] (Fig. 1A) monitoring the release of Gβγ from the heterotrimeric G (Gαβγ) protein upon activation by monitoring the occurrence of BRET between Gβ_1_γ_3_-RlucII, the energy donor, which interact with GRK2 fused to the energy acceptor GFP10 (GRK2-GFP10). Using this assay, we tested the ability of CXCL12 to promote ACKR3-mediated activation of 13 human and 7 mouse G proteins. As can be seen in Fig. 1C, D and Additional file 1: Figure S1B, C, none of the G proteins could be activated by ACKR3, confirming previous studies reporting lack of G protein engagement by the atypical chemokine receptor [12, 13, 15, 37]. Cells transfected with the delta opioid receptor (DOR) were used as a positive control for the assay and indeed showed robust G protein activation for the Gi/Go family members upon treatment with Met-Enkephalin (Fig. 1B and Additional file 1: Figure S1A).

We then examined the affect of the Venus tag on ACKR3 membrane localization and agonist induced trafficking. To this aim, we added a Flag epitope to the N-terminus of ACKR3 and ACKR3-Venus, and labeled the receptors at 37 °C with Flag antibody conjugated to a fluorphore (M1-Alexa-647). This strategy enables the labeling of the membrane surface population of receptors only. Confocal microscopy images show that both ACKR3 and ACKR3-Venus receptor cluster at the plasma membrane (Fig. 1E). To examine ligand-induced trafficking, we transfected cells with ACKR3-Venus without the Flag and treated cells with 300 nM CXCL12 or vehicle. We observed time-dependent trafficking of ACKR3-Venus following CXCL12 treatment but not vehicle (Fig. 1F). CXCL12 scavenging was also examined using a selective ligand for ACKR3 {Ameti, 2018 #4373} fluorescently conjugated CXCL11-12-Alexa647 and similar localization of CXCL12 was observed in ACKR3 and ACKR3-Venus cells (Additional file 1: Figure S1D). These results suggest that the addition of the Venus on the receptor C-terminus does not impair membrane localization or trafficking of the receptors.

We then used a βarrestin ebBRET biosensor assay [34] (Fig. 1G, donor- βarrestin2-RlucII; acceptor rGFP-CAAX) to assess βarrestin2 recruitment to tagged and untagged ACKR3 at the plasma membrane. We observed no statistical difference in concentration-dependent βarrestin2 recruitment to ACKR3 and ACKR3-Venus upon CXCL12 stimulation (EC_50_ 1.9 nM vs. 2.2 nM). The Venus tag did not contribute to the BRET signal as we detected similar BRET signals in the absence of the acceptor rGFP-CAAX as compared to untagged ACKR3 (Fig. 1H). We therefore conclude that the addition of the C-terminally fused Venus did not affect the signaling properties of ACKR3 which fails to activate G proteins but efficiently recruits βarrestin2 upon agonist stimulation.

### ACKR3-Venus as a biosensor to monitor βarrestin recruitment

**Fig. 2:**
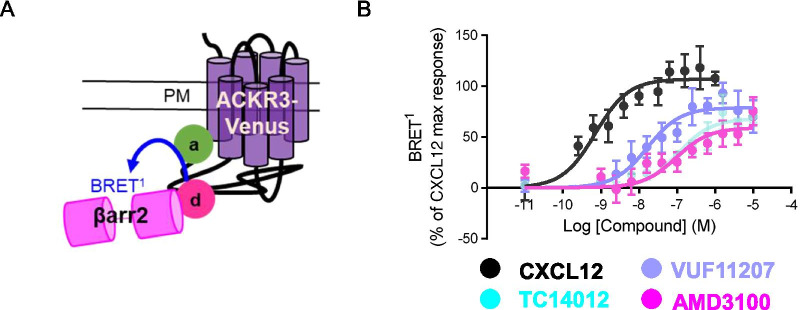
Ligand profiling of βarrestin2 recruitment to ACKR3-Venus in HEK-293 cells. **A** Schematic representation of the BRET^1^ biosensor monitoring the interaction between βarrestin2-RlucII (BRET^1^ sensor donor) and Venus-tagged ACKR3 (BRET^1^ sensor acceptor) [79]. **B** Cells transfected with ACKR3-Venus and βarrestin2-RlucII were exposed for 10 min to increasing concentrations of the indicated compound. Data are expressed as % of CXCL12 maximum response (Mean ± SEM) of 3–5 independent experiments

ACKR3 binds to two endogenous chemokine ligands CXCL12 and I-TAC/CXCL11 which are also ligands to CXCR4 and CXCR3, respectively [6, 7]. Several synthetic compounds have also been developed for ACKR3 as research tools [27]. The small-molecule CXCR4 selective antagonist AMD3100, a once promising anti-HIV drug, currently approved for clinical use for lymphoma and multiple myeloma (for a review see [38]), has also been shown to be an allosteric ACKR3 agonist at high concentrations [39] activating βarrestin recruitment and increasing CXCL12 binding to ACKR3. VUF11207, is a high-affinity selective ACKR3 small molecule agonist reported to recruit βarrestin [40]. Finally, TC14012 is a peptidomimetic inverse agonist of CXCR4 and has been reported to be a ACKR3 agonist with high potency for βarrestin recruitment [41]. Thus, we next asked whether ACKR3-Venus could be used directly, as an acceptor, in a βarrestin recruitment BRET^1^ assay (Fig. 2A, donor—βarrestin2-RlucII; acceptor—ACKR3-Venus) to discern the pharmacological profiles of these four ACKR3 agonists, CXCL12, AMD3100, VUF11207 and TC14012 in HEK-293 cells (Fig. 2B). ACKR3-Venus stimulation by all compounds promoted the recruitment of βarrestin2 with the following order of potency, from high to low, CXCL12, VUF11207, AMD3100 and TC14012 (Table 1). When compared to CXCL12, the 3 synthetic compounds acted as partial agonists for βarrestin recruitment with efficacies of 79%, 68% and 59% for VUF11207, TC14012 and AMD3100 respectively (Table 1). These data show that ACKR3-Venus is a functional receptor that can be used as a biosensor in a receptor proximal assay that directly monitors ACKR3 signaling activities for various ligands indicating that the KI mice expressing this construct could be used to study the activity of ACKR3-targeting drugs.

**Table 1:** βarrestin2 recruitment to ACKR3-Venus in HEK-293 cells

Compound	Emax (%)	LogEC50
CXCL12	100.0 ± 5.6	− 9.15 ± 0.15
VUF11207	73.8 ± 5.6	− 7.78 ± 0.18
TC14012	63.6 ± 4.6	− 6.97 ± 0.14
AMD3100	55.2 ± 6.2	− 7.02 ± 0.23

Cells were exposed for 10 min to increasing concentrations of the indicated compound. Emax values are expressed as % of the maximal response induced by CXCL12. Potency is expressed as LogEC_50_. Data are the Mean ± SEM of 3–5 independent experiments (Fig. 2B)

### Creation of Ackr3-Venus knock-in mice

**Fig. 3:**
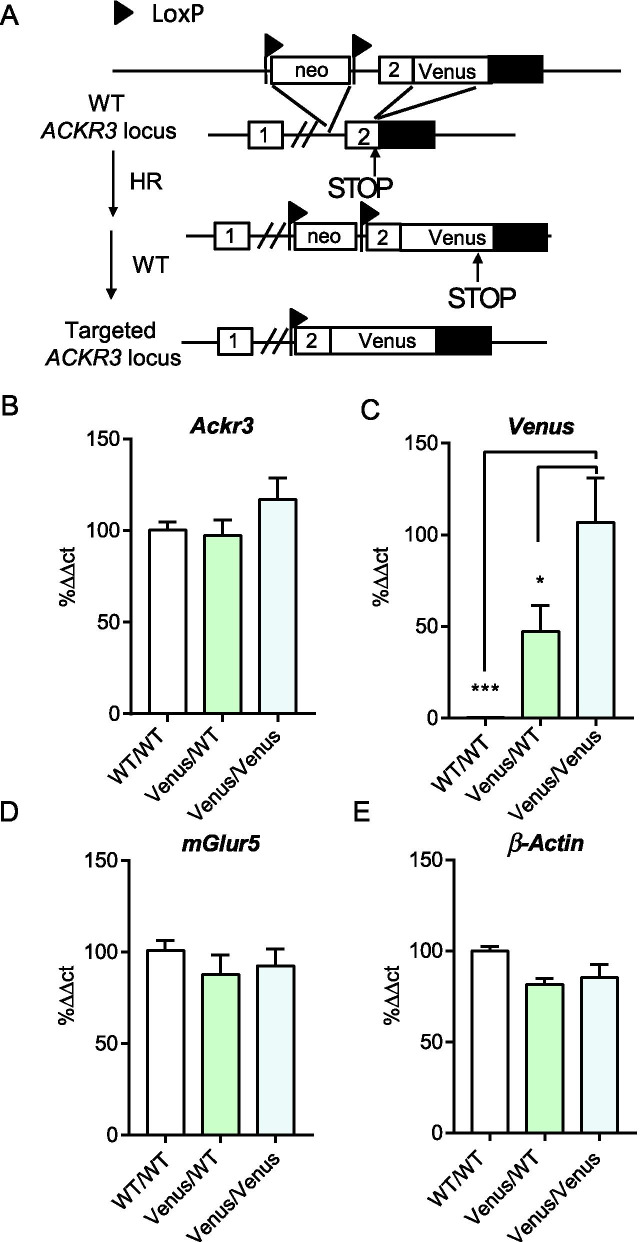
*Ackr3-Venus* knock-in mouse generation. **A** The scheme shows the strategy used to generate *Ackr3-Venus* knock-in mice (see *methods*). Briefly, the intron 1- exon 2 of *Ackr3* was replaced with a targeted fragment containing *Ackr3*-fused to Venus at C-tail. LoxP sites are shown by black flags, closed box is 3′ UTR. **B**–**E** Evaluation of *Ackr3-Venus* transcript levels from RNA extracts of whole mouse brain. Real-time qPCR results show expression levels of the following transcripts *Ackr3* (**B**), *Venus* (**C**) *mGluR5* (**D**), and *β-actin* (**E**), *in Ackr3*^WT/WT^, *Ackr3*^*Venus/WT*^, and *Ackr3*^*Venus/Venus*^ (n = 3–6 animals per group). The ΔΔCT in percent of WT is shown for each transcript except Venus where KI was used for normalization. Significance is shown as *p < 0.05 and ***p < 0.001 in a one-way ANOVA with Dunn’s multiple comparison’s test

Using a homologous recombination approach that was successfully used for other GPCRs [42–45] we next generated a KI mouse with Venus fused to the C-tail of *Ackr3*, which we refer to as *Ackr3-Venus* mice (Fig. 3A). We first examined the mRNA transcript level of *Ackr3* in whole brain and observed no significant change at the transcript level of homozygous *Ackr3*^*Venus/Venus*^ or heterozygous *Ackr3*^*Venus/WT*^ as compared to wild-type *Ackr3*^*WT/WT*^ (Fig. 3B). However, as expected, the fluorescent protein, *Venus,* transcript was gene-dose-dependently increased, with none detected in *Ackr3*^*WT/WT*^ animals (Fig. 3C). Notably, similar levels of another GPCR, *mGlur5*, and a housekeeping gene, *β-actin*, were observed across all three genotypes (Fig. 3D, E, respectively) suggesting that the observed alterations in *Venus* transcripts were specific to the genetic manipulations performed.

### Mapping of ACKR3-Venus expression in peripheral organs of knock-in mice

**Fig. 4:**
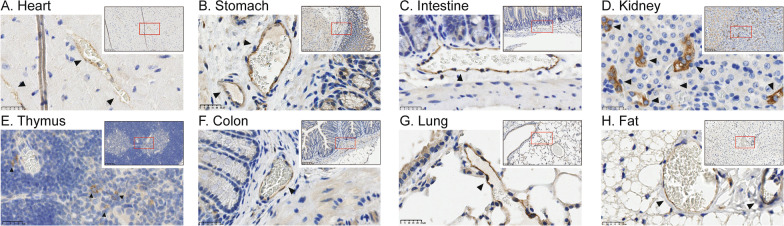
ACKR3-Venus expression in peripheral mouse organs. Representative images of amplified ACKR3-Venus expression from eight peripheral tissues prepared from adult (P56 to P72) female and male mice. Organs from *Ackr3*^*Venus/Venus*^ mice were sectioned transversely (4 µm), and the expression of Venus was revealed with a mouse Venus (GFP) antibody (brown) and counterstained with hematoxylin labeling of cell nuclei (blue). Shown are representative images of organs where ACKR3-Venus is observed **A** heart (male) **B** stomach (female) **C** intestine (male) **D** kidney (female) **E** Thymus (female) **F** colon (female) **G** lung (male) **H** Fat (male). Arrowheads are located at regions with ACKR3-Venus expression. A red box on the inset indicates where the higher magnification image was taken. Organ sections were imaged on the Hamamatsu’s NanoZoomer^®^ Digital Pathology system 2HT with 80× (large image) and 20× (inset) objectives. Scale bar is 25 µm

To map the expression of ACKR3-Venus in peripheral organs, 13 tissues were dissected from adult *Ackr3*^*Venus/Venus*^ and *Ackr3*^*WT/WT*^ animals and used for immunohistochemistry against the Venus tag. ACKR3-Venus expression was restricted to vascular endothelium in most organs such as heart, stomach, intestine, colon, and lung (Fig. 4A–C, F–G). However, a high expression of the receptor was found in kidney medulla (Fig. 4D) and in spleen (Additional file 1: Figure S3), particularly in red pulp (Additional file 1: Figure S3A, E) and marginal zone B cells (Additional file 1: Figure S3D, H) which agrees with previous studies on the expression of ACKR3 in spleen [18, 46]. ACKR3-Venus expression was also observed at low levels in thymus medulla (Fig. 4E) and fat (Fig. 4H). ACKR3-Venus expression was not detected in the *Ackr3*^*WT/WT*^ females (Additional file 1: Figure S4A–N) or males (Additional file 1: Figure S6A–O). Interestingly, we found ACKR3-Venus expression in *Ackr3*^*Venus/Venus*^ animals to be different in males and females for some tissues, as females (Additional file 1: Figure S4, S5) presented higher receptor expression in the kidney medulla (Additional file 1: Figure S4J) and liver (Additional file 1: Figure S4E) as compared to males (Additional file 1: Figure S6, S7J, E). The complete distribution of ACKR3-Venus across these peripheral tissues in female and male *Ackr3*^*Venus/Venus*^ animals is summarized in Table 2, demonstrating the utility of these animals for detecting ACKR3 in peripheral tissues.

**Table 2:** ACKR3-Venus expression in the adult mouse peripheral organs

	Female	Male
Digestive system		
Stomach	+ +	+ +
Intestine	+ +	+ +
Liver	+	n.a
Pancreas	+ +	+ +
Colon	+ +	+ +
Reproductive system		
Uterine Horn	+ +	n.a
Testes	n.a	+ +
Seminal vesicles	n.a	+ +
Lymphatic system		
Spleen	+ + + +	+ + +
Thymus	+	+
Fat		
White fat	+	+
Cardio-respiratory system		
Heart	+	+
Lung	+ +	+ +
Urinary system		
Kidney	+ + +	+

Amplified ACKR3-Venus expression was assessed in adult (P56-P72) animals. Thirteen *Ackr3*^*Venus/Venus*^ mouse organs were obtained from females and males. Tissues were immunostained to amplify the Venus tag. Expression in *Ackr3*^*Venus/Venus*^ were compared to any background staining observed in *Ackr3*^*WT/WT*^ (n = 3) (Additional file 1: Figure S4–S7) organs. The ACKR3 organ expression of *Ackr3*^*Venus/Venus*^ are ranked as follows: low (+), moderate (+ +), high (+ + +), very high (+ + + +) expression. This analysis was only qualitative, and no statistical analysis has been done

### ACKR3-Venus intrinsic vs. amplified expression in the brain of knock-in mice

**Fig. 5:**
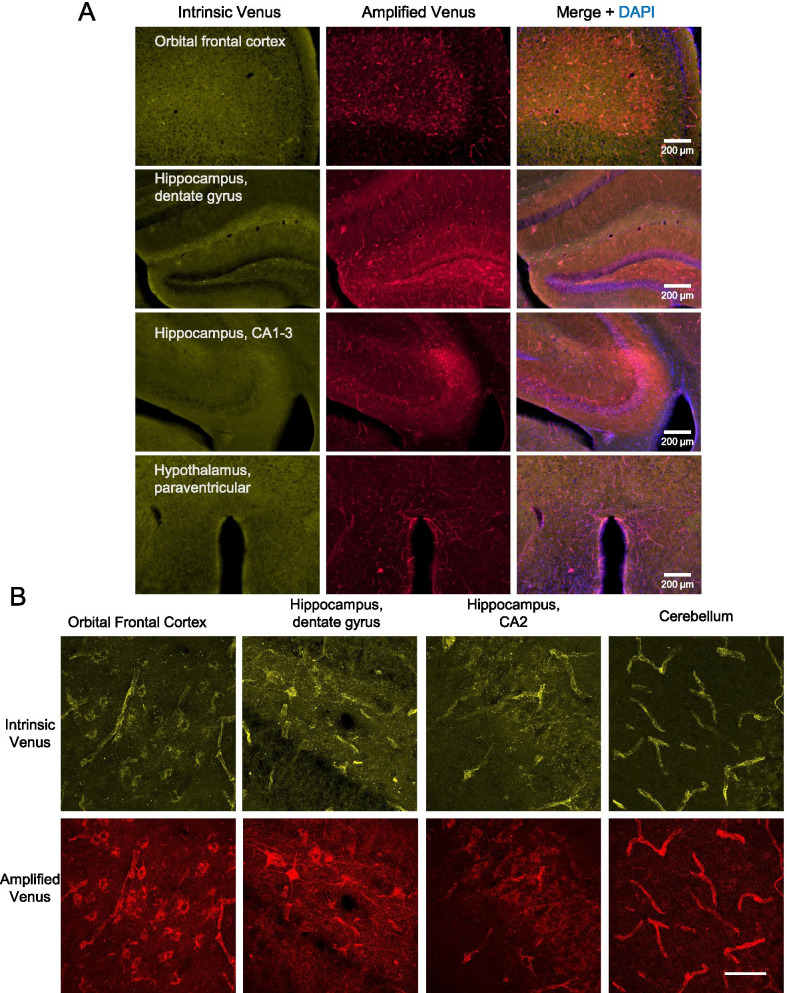
Intrinsic and amplified regional and cellular expression of ACKR3-Venus in the adult mouse brain. Brain sections were prepared from *Ackr3*^*Venus/Venus*^ mice. To allow for a comparison between the intrinsic unamplified (yellow) and the amplified (red) Ackr3-Venus signal, we amplified Venus in the red channel. **A** Representative images of brain regions with ACKR3-Venus expression (intrinsic, yellow; amplified, red). DAPI was used to stain cell nuclei. Images were taken on Olympus IX73 epifluorescent microscope, 10 × objective. Scale bar is 200 µm. **B** To observe ACKR3-Venus at the cellular level, we imaged the brain sections from *Ackr3*^*Venus/Venus*^ mice on a laser scanning confocal microscope (Olympus FV1200) with a 60 × oil objective. Shown are representative brain areas with distinct expression patterns. Scale bar is 50 µm

Epifluorescent microscopy images of the intrinsic ACKR3-Venus signal revealed cerebral vasculature throughout the brain as shown in cortex, septum, caudate putamen, hippocampus, and hypothalamus (intrinsic Venus) that was more easily detectable when amplified with anti-Venus (amplified Venus) (Fig. 5A). The ACKR3-Venus signal was absent from *Ackr3*^*WT/WT*^ brain sections (Additional file 1: Figure S8). With confocal microscopy and higher magnification, ACKR3-Venus intrinsic signal was easily detectable in the same cells as the amplified signal (Fig. 5B). ACKR3-Venus positive blood vessels were observable throughout the imaged sections as seen in areas such as OFC, HPF and cerebellum. In the OFC and HPF discrete neuronal-looking cell populations were noticeable. Together, these data show how traceable ACKR3-Venus expression is, by either the direct observation of intrinsic Venus signal or by antibody amplification of the Venus tag in brain tissue from *Ackr3*^*Venus/Venus*^ mice.

### Mapping of ACKR3-Venus expression in whole brain of knock-in mice

**Fig. 6:**
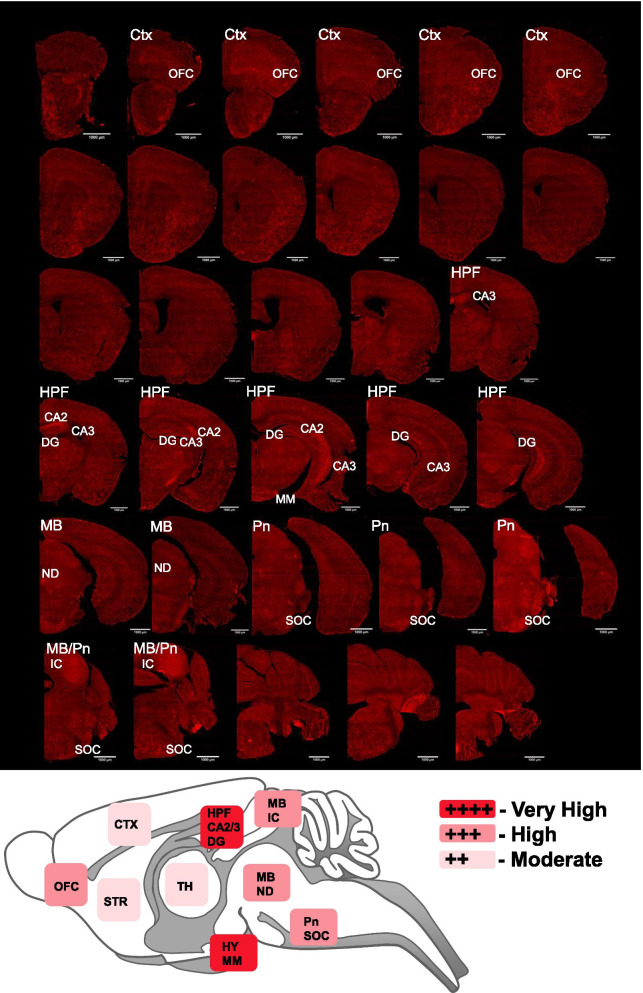
ACKR3-Venus whole brain distribution in the adult mouse brain. Amplified ACKR3-Venus expression was examined throughout the adult (P56) mouse brain (Table 3). Brains from *Ackr3*^*Venus/Venus*^ mice (n = 4) were sectioned coronally on a cryostat (30 µm). Venus fluorescence was amplified with Anti-GFP (red), and slides were scanned on a slide scanner widefield microscope (Olympus). Images were scanned with a 10x objective. Top—Annotated regions shown here in red, contained the highest enrichment of ACKR3-Venus expression. Bottom—A scheme demonstrates the range of observed ACKR3-Venus expression across brain regions. *Ctx* cortex, *OFC* orbital-frontal cortex, *DG* dentate gyrus, *HPF* hippocampal formation, *MB* midbrain, *Pn* pons, *Hy* Hypothalamus, *IC* inferior colliculus, *MM* medial mammillary, *STR* striatum, *SOC* superior olivary complex, *TH* Thalamus, *ND* nucleus of Darkschewitsch. Scale bar is 1000 µm

Adult *Ackr3*^*Venus/Venus*^ mouse brain was then used to map ACKR3-Venus expression in the central nervous system. We first amplified ACKR3-Venus using an anti-GFP antibody in the red channel to be able to evaluate intrinsic Venus signal separately. At macrolevel, the brain areas with the highest enrichment of ACKR3-Venus include the orbitofrontal cortex (OFC), hippocampal (HPF) regions—CA2, CA3, dentate gyrus (DG), hypothalamic mammillary nucleus, midbrain nucleus of Darkschewitsch, inferior colliculus, and pons—superior olivary complex (Fig. 6). We ranked ACKR3-Venus expression across the whole brain and compiled a table of 112 selected brain areas that express ACKR3-Venus to varying degrees (Table 3). Amplified ACKR3-Venus was observable throughout the brain either on brain vasculature or in discrete cell populations. We next examined which cell sub-types expressed ACKR3-Venus receptors.

**Table 3:** ACKR3-Venus mapping in the adult mouse brain

Cortex		Cortical Subplate		Midbrain cont	
Agranular Insular	+ +	Bed Nucleus of the stria Terminalis	+	IPN Dorsolateral Subnucleus	+ +
Auditory	+ +			IPN, Lateral Subnucleus	+ +
Cingulate	+ +	Thalamus		IPN, rostral Subnucleus	+ +
Dysgranular Insular	+ +	Anteromedial nucleus	+ +	Interstitial nucleus of Cajal	+ +
Ectorhinal	+ +	Central Medial	+ +	Periaqueductal Gray	
Entorhinal	+ +	Geniculate	+ +	Dorsomedial	+ +
Granular Insular	+ +	Ethmoid	+	Supraoculomotor	+ +
Motor	+ +	Interanteromedial	+ +	Posterior commissure nucleus	+
Orbital Frontal	+ + +	Intergeniculate	+	Pretectal nucleus	+ +
Parietal Association	+ +	Intermediodorsal	+ +	Raphe nucleus	
Piriform	+ +	Laterodorsal	+ +	Dorsal, Caudal	+
Primary Somatosensory	+ +	Lateral Posterior, Mediocaudal	+	Dorsal, Ventral	+ +
Secondary Somatosensory	+ +	Mediodorsal	+ +	Median	+ +
Retrosplenial	+ +	Medial Geniculate	+ +	Substantia Nigra Compact part	+ +
Temporal Association	+ +	Paracentral	+ +	Superior Colliculus	
Visual	+ +	Paraventricular	+	Intermediate White layer	+ +
Olfactory Bulb	+ +	Posterior	+	Optic Nerve layer	+ +
		Rhomboid	+ +		
Cortical Subplate		Submedius	+ +	Pons	
Basal Lateral Amygdala	+	Subparafascicular	+	Dorsomedial Tegmental area	+
Claustrum	+ + +	Ventral	+ +	Laterodorsal Tegmental nucleus	+ +
		Epithalamus		Lateral Lemniscus	+ + +
Striatum		Lateral Habenula	+ +	Locus Coeruleus	+
Amygdala		Medial Habenula	+ +	Motor Trigeminal nucleus	+ + +
Central				Tensor Tympani part	+ +
Lateral	+ +	Hypothalamus		Parabrachial nucleus	+
Medial	+ +	Arcuate	+	Pontine nuclei	+ + +
Extended	+	Dorsomedial	+	Pontine Reticular nucleus	+
Intercalated	+ +	Lateral Mammillary	+ + + +	Posterodorsal Tegmental nucleus	+ +
Medial Anterodorsal	+ +	Preoptic	+	Principal Sensory Trigeminal nucleus	+ + +
Lateral Septum		Lateral	+	Reticulotegmental nucleus	+ +
Dorsal	+ +	Median Eminence	+	Superior Olive	+ + +
Ventral	+	Paraventricular	+ + +		
Nucleus Accumbens	+	Perifornical	+	Medulla	
		Retrochiasmatic	+	Cochlear nucleus	+ + +
Hippocampus		Septohypothalamic	+	Cuneiform nucleus	+
CA2	+ + + +	Striohypothalamic	+	Efferent Vestibular nucleus	+ +
CA3	+ + + +	Subparaventricular	+	Gigantocellular Reticular nucleus	+ +
Polymorph layer Dentate Gyrus	+ + + +	Suprachiasmatic	+	Medial Vestibular nucleus	+ +
Fasciola cinereum	+ + + +	Supraoptic	+ + +	Parapyramidal nucleus	+
Fimbria	+	Ventromedial	+	Parvicellular Reticular nucleus	+ +
Parasubiculum	+ +				
Postsubiculum	+ +	Midbrain		^Cerebellum^	
Presubiculum	+ +	Darkschewitsch nucleus	+ + +	4th/5th Cerebellar lobules	+ +
Pyramidal cell layer	+ + + +	Inferior colliculus	+ + +	Crus 1 Cerebellar lobules	+ +
		Interpeduncular Nucleus (IPN)	+	Flocculus	+ +

Amplified ACKR3-Venus expression was mapped throughout the adult (P56) *Ackr3*^*Venus/Venus*^ mouse brain. If ACKR3-Venus expression was not observed in a region or was not observed at the same expression level consistently across animals (n = 4), the region was not included in this table. Expression in *Ackr3*^*Venus/Venus*^ (n = 4) were compared to negligible background staining observed in *Ackr3*^*WT/WT*^ (n = 4) (Additional file 1: Figure S8). The brain regions included in this table are derived from The Mouse Brain in Stereotaxic Coordinates, Paxinos and Franklin, Second Edition, 2001. The expression of ACKR3-Venus across brain regions is ranked as follows: low (+), moderate (+ +), high (+ + +), very high (+ + + +) expression. This analysis was qualitative, and no statistical analysis has been done

### ACKR3-Venus is expressed in GABAergic interneurons of the hippocampus

**Fig. 7:**
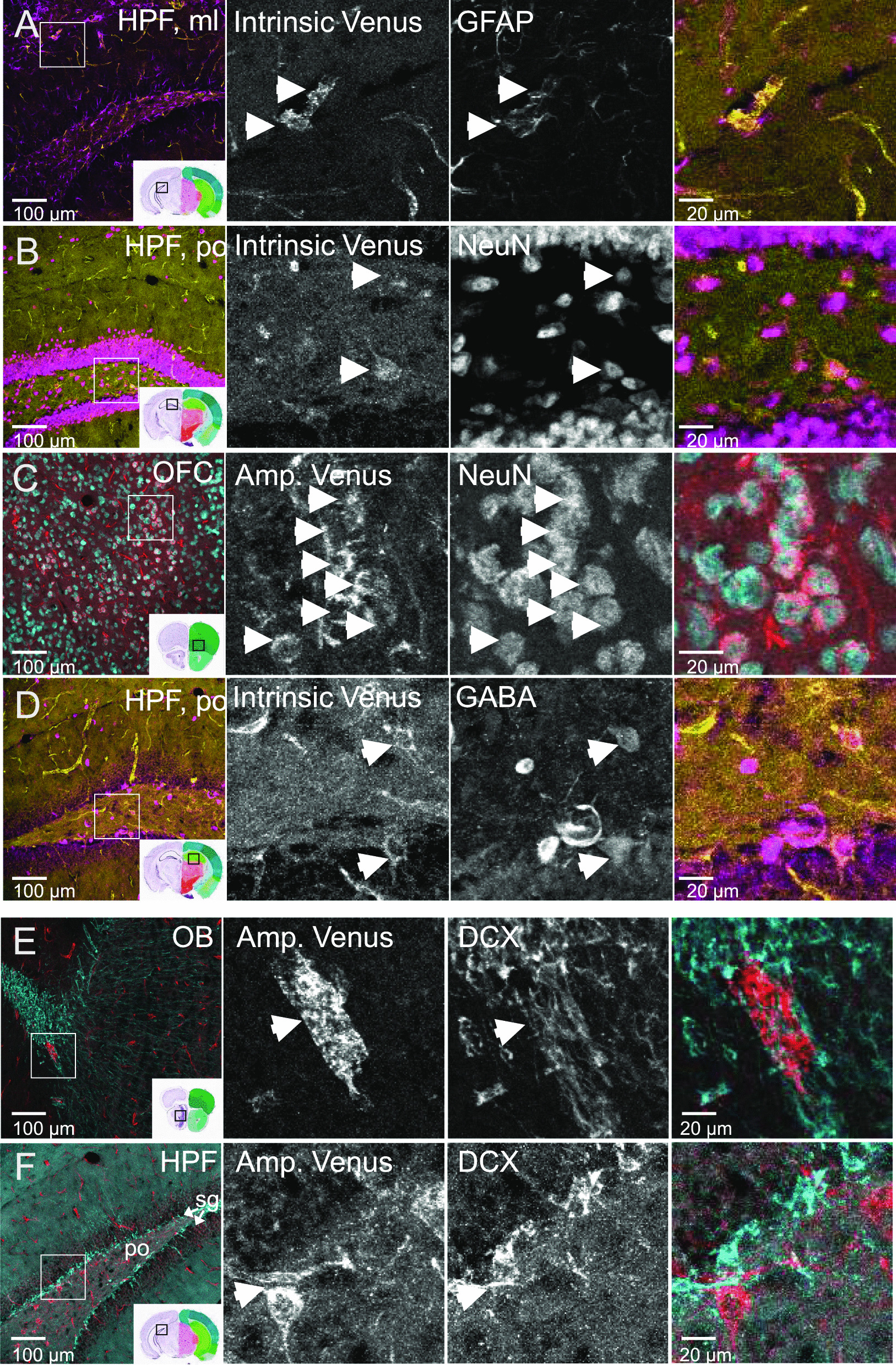
ACKR3-Venus expression in glial and neuronal cell-types in the adult mouse brain. ACKR3-Venus was examined at the cellular level using cell subtype markers. Shown are representative images for intrinsic (yellow) or amplified anti-Venus (red) with the cell sub-type marker (magenta, if imaged together with intrinsic Venus (**A**, **B**, **D**) or cyan, if imaged with amplified (Amp.) Venus (**C**, **E**, **F**)]. In each row, the first image is 20 × magnification to show brain area and the following three images are at 5 × higher magnification to show cellular level. The middle two images in black and white indicate single antibody channel and the color images are merged images of both channels. **A** Astrocytes identified by anti-GFAP (magenta) are shown together with intrinsic Venus (yellow) in the molecular layer (ml) of the hippocampus (HPF). **B** Neurons identified by anti-NeuN (magenta) are shown together with intrinsic Venus (yellow) in the polymorphic layer (po) of the dentate gyrus in the HPF. **C** Neurons identified by anti-NeuN (cyan) are shown together with amplified Venus (red) in the orbital frontal cortex (OFC). **D** GABAergic interneurons identified by anti-GABA (magenta) are shown together with intrinsic Venus (yellow) in the po of the dentate gyrus in the HPF. **E**, **F** Newly generated neurons identified by anti-DCX (cyan) are shown together with amplified Venus (red) in the olfactory bulb (**E**) and at the intersection of the granule cell layer (sg) and po of the dentate gyrus in the HPF (**F**). White boxes (left column) correspond to the 5 × magnified images. Insets contain mouse brain reference atlas images from Allen Brain Atlas, with the region locations outlined by black boxes. White arrowheads indicate cells that are co-labeled, **A**–**D** or label adjacent, **E**, **F**. Brain sections from *Ackr3*^*Venus/Venus*^ mice were imaged on an Olympus FV1200 laser scanning confocal microscope, 20 × objective. Scale bar of 20 × image is 100 µm (1st column) and 20 µm (2nd–3rd columns)

We next wanted to identify the cellular sub-type for ACKR3-Venus in selected brain areas of *Ackr3*^*Venus/Venus*^ mice using confocal imaging. We first examined astroglial cells by staining brain sections with glial fibrillary acid protein (GFAP), the main astrocyte-specific intermediate filament protein [47]. GFAP positive cells were easily detectable throughout the brain but sparse co-labeling was observed for GFAP and intrinsic fluorescence of ACKR3-Venus receptors, such as seen in the molecular layer of the hippocampus (Fig. 7A). Additional glial cell-type staining, including CNPase, 2′, 3′-cyclic nucleotide 3′-phosphodiesterase for mature oligodendrocyte and schwann cell identification (data not shown) and NG2, a membrane chondroitin sulfate proteoglycan, expressed on oligodendrocyte precursor cells [48] (Additional file 1: Figure S9) both failed to co-label with ACKR3-Venus positive cells. Whereas the NeuN labelling of neuronal nuclei [49, 50] co-labeled much of the intrinsic ACKR3-Venus neurons in the hippocampus, polymorphic layer (Fig. 7B) and many of the amplified ACKR3-Venus positive neurons in the OFC (Fig. 7C). Finally, we identified the sub-type of neurons in the polymorphic layer of the hippocampus DG as primarily GABA positive (Fig. 7D). Altogether, these experiments show that ACKR3-Venus localizes to neurons a subset of which are hippocampal GABAergic interneurons.

### ACKR3-Venus is expressed adjacent to DCX + neuroblasts of SVZ/RMS and DG

Doublecortin (DCX) microtubule-associated protein is a marker of both embryonic corticogenesis and adult neurogenesis [51] which primarily occurs in the subventricular zone (SVZ)/rostral migratory stream (RMS)/olfactory bulb and the dentate gyrus of the hippocampus [52]. Since we observed discrete neuronal populations expressing ACKR3-Venus in those areas, we co-labeled the sections with anti-DCX. We detected DCX neuroblasts that migrate in chain-like [53] clusters alongside vascular cells on ACKR3-Venus illuminated blood vessels along the SVZ/RMS route to the olfactory bulb (Fig. 7E) and DCX + cells in the granule cell layer of the dentate gyrus which were visibly adjacent to ACKR3-Venus neurons of the hippocampus (Fig. 7F). These data suggest that ACKR3 is not co-expressed in neuroblasts but localizes to cells proximal to neuroblasts.

### ACKR3-Venus is expressed on vimentin + and CD31 + cerebral vasculature

**Fig. 8:**
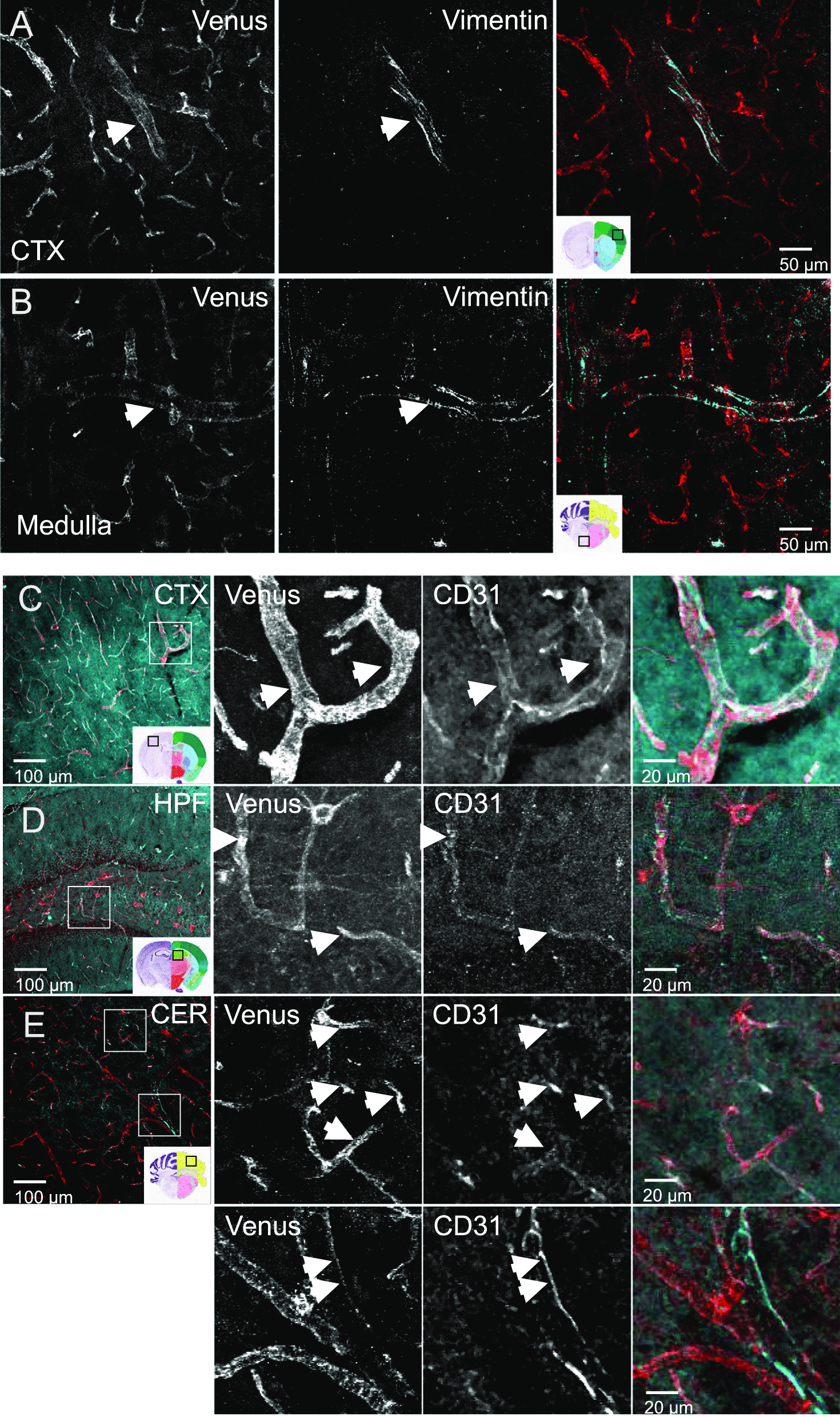
ACKR3-Venus vascular cellular expression in the adult mouse brain. Shown are representative images of brain sections from *Ackr3*^*Venus/Venus*^ for ACKR3-Venus amplified expression (red) and vascular cell markers, vimentin (**A**, **B**) or CD31 (**C**–**E**) in cyan. Representative images show vimentin stains larger blood vessels in the cortex (CTX, **A**) and medulla (**B**). Anti-CD31 was used to mark endothelial cells (cyan) shown are representative images from the CTX (**C**), hippocampus (HPF, **D**) and cerebellum (CER, **E**) with amplified Venus. Images were taken on Olympus FV1200 Laser scanning confocal microscope 20 × objective. White arrowheads indicate cells that are co-labeled, **A**–**E**. Scale bar is **A**, **B**, 50 µm or **C–E,** 100 µm (1st column) and 20 µm (2nd–3rd columns)

Vimentin is an intermediate filament protein found in many types of immature cells throughout the body, including primitive neuroepithelial cells. It is regarded as a neural stem cell marker but is also expressed in several other mature cell types in the CNS: endothelial cells of blood vessels, smooth vascular musculature and fibroblasts [54]. Therefore, we next examined the expression of ACKR3-Venus on vimentin + cells. We found ACKR3-Venus receptors expressed on cells co-labeled with vimentin + large blood vessels in areas including the cortex (Fig. 8A) and the medulla (Fig. 8B). Together, these data show that ACKR3-Venus expression is localized to immature endothelial cells on large vimentin + blood vessels.

CD31 or platelet endothelial cell adhesion molecule-1 (PECAM-1), is found in large quantities on the surface of endothelial cells [55, 56]. ACKR3 has been reported to localize to endothelial cells in cultured human cells [57] and in vivo in mouse brain [30]. While abnormal ACKR3 expression on endothelial cells has been shown to negatively regulate homeostatic endothelial functions [58]. We next examined ACKR3 expression in endothelial cells using anti-CD31 labeling in *Ackr3*^*Venus/Venus*^ brain sections. We observed co-labeling along both macro and micro-blood vessel walls throughout the brain, such as in cortex (Fig. 8C), hippocampus (Fig. 8D), and cerebellum (Fig. 8E). These findings suggest that ACKR3-Venus is localized to CD31 + mature endothelial cells on mouse brain vasculature.

## Discussion

ACKR3 is an atypical chemokine-like receptor that binds CXCL12 a ligand with critical roles in angiogenesis, development, inflammation, immune challenges, and cancer [57]. Here, we examined the expression of ACKR3 in peripheral organs and brain using the first ever engineered fluorescent knock-in (KI) mouse for direct detection of ACKR3, *Ackr3-Venus*. Here, we show that the modified fluorescent receptor retains hallmarks of the native receptor, based on BRET biosensor signaling assays in HEK-293 cells and already known expression sites from existing reports. Thus, the *Ackr3-Venus* KI mouse line generated here is a valuable novel tool that will be useful to study ACKR3 biology by directly monitoring receptor trafficking and interaction with complementary fluorescent fusion proteins.

### ACKR3 signaling

In agreement with previous literature [12], we found ACKR3-Venus is a βarrestin biased receptor. Activation of ACKR3-Venus by CXCL12 in a BRET assay measuring G protein activation for 13 human and 7 mouse G proteins found no response for any of the G proteins tested. One report has shown that in cultured primary astrocytes and human glioma cells, CXCL12 induced ACKR3 mediated increases of intracellular calcium and pertussis toxin sensitive ERK and AKT signaling suggesting ACKR3 activates Gi/o [59]. However, we did not see any G protein activity for ACKR3 in our assay suggesting that there may be components required for these ACKR3 signaling activities that are cell-type specific. To our knowledge, this is the first report examining ACKR3 signaling across such a wide selection of mouse and human G proteins.

We also examined βarrestin recruitment by monitoring βarrestin recruitment directly to the receptor in a BRET assay where ACKR3-Venus is the acceptor (Fig. 2B) to determine the agonist profile for 4 compounds, CXCL12, TC14012, VUF11207 and AMD3100. The cognate ligand CXCL12 was the most potent in agreement with previous reports [41]. All 4 agonists showed activities that could be differentiated using ACKR3-Venus as the biosensor. Importantly, we observed distinct profiles for all four of the agonists tested in this assay suggesting that the ACKR3-Venus mouse can be used to evaluate the pharmacology of drugs targeting this atypical receptor.

### ACKR3 expression in peripheral tissues

In male and female animals, we observed ACKR3-Venus on vascular endothelium to varying degrees in heart, stomach, intestine, pancreas, kidney, spleen, thymus, fat, colon, lung, and reproductive organs (Fig. 4, Additional file 1: Figures S2–S7 and Table 2). Our observations are in general agreement with the described expression of *Ackr3* transcripts in mouse [6, 60] and human [6, 19, 60] and ACKR3 tissue distribution shown by reporter mouse lines in which the ACKR3 promoter induces LacZ expression allowing for ACKR3 + cells to be detected by β-galactosidase [6, 19, 29, 31]. *Ackr3* mRNA has been reported in human liver, but we observed negligible ACKR3-Venus in this organ and only in female mice (Additional file 1: Figure S4, S5 and Table 2). The lack of ACKR3-Venus in liver may be due to differences between species or developmental timepoints, ACKR3 has been shown to bind to CXCL12 transiently at E11 and E13 but not E15 and E17 in fetal mouse liver [6]. ACKR3 has been reported as a potential therapeutic target for peripheral tissue disease such as cancer, cardiovascular diseases, and inflammatory processes [61–63] and the *Ackr3-Venus* KI mouse generated here will be instrumental in the ongoing research efforts to understand ACKR3 signaling in peripheral tissues [64] and contributions to disease.

### ACKR3 expression in the brain

Using *Ackr3-Venus* KI animals, we detected ACKR3-Venus without any signal amplification in the following brain areas, orbital frontal cortex, hippocampus, hypothalamus and, in vasculature throughout the brain (Figs. 5, 6). Our observations agree with reports of *Ackr3* transcript expression at high level in hippocampus, moderate level in cortex and brain vasculature of naïve rats [65]. Our data is also in agreement with the ACKR3 mouse reporter datasets described earlier (see “Introduction”) in which ACKR3 + cells were identified in cerebral cortex [29] and olfactory bulb, cerebral cortex, hippocampus, subventricular zone, hypothalamus and cerebellum with notable expression in cerebral blood vessels [30]. In summary, at whole region level, ACKR3-Venus animals have expected ACKR3 CNS distribution, and these animals can therefore be used to detect the receptor throughout the mouse brain (Table 3).

An advantage of the knock-in animals that we have characterized here, is the ability to identify cell populations by direct observation of the receptor itself, ACKR3-Venus. We examined neuronal cell-types with immunolabeling cell-type markers. We identified sparse ACKR3-Venus on GFAP + cells (Fig. 7A) in the hippocampus suggesting that there may be a select group of astrocytes or brain region which expresses ACKR3 on astrocytes. In the future, primary astrocytes may be cultured from these mice to investigate the proposed ACKR3 mediated G protein signaling [59] from these cells.

In the cortex and hippocampus, we found ACKR3-Venus to co-stain with NeuN labeled neurons (Fig. 7B, C). We identified dentate gyrus hippocampal ACKR3-Venus neurons to be GABAergic interneurons as they co-localized with GABA (Fig. 7D). At embryonic stages, ACKR3 expression is promoted by transcription factors which regulate interneuron development [66] and its involvement has been shown in the regulation of interneuron migration [21, 23]. In the adult, ACKR3 has been identified in pyramidal cells [65], dentate gyrus (DG) granule cells and is often reported in GABAergic interneurons suggesting that ACKR3 may mediate CXCL12 in several brain areas that could impact GABAergic and glutamateric signaling. Notably, silencing ACKR3 in the hippocampal DG region has been shown to have an antiepileptic effect in a mouse model of epilepsy [24] suggesting important neuronal regulatory functions for ACKR3. Here, we detected intrinsic ACKR3-Venus on the hippocampal GABAergic interneuron population, future neuronal phenotyping of ACKR3-Venus receptor expression on cells from ACKR3-Venus animals may reveal other ACKR3 neuronal subtypes shedding light on ACKR3 neurobiology.

We observed DCX + cells (Fig. 7E, F) proximal to ACKR3-Venus in the hippocampus. Neural progenitor cell survival has been demonstrated in cultures to be mediated via ACKR3/CXCR4 endocytosis [67]. Also, ACKR3 has been shown to be expressed in migrating cortical neurons [68] that express DCX. Furthermore, a role for ACKR3 in neurogenesis by mediating CXCL12-CXCR4 axis in migrating cortical interneurons in the embryonic brain of mice has recently been shown [21, 25]. ACKR3 therefore is a potential regulator of cortical composition and inhibitory circuits.

We detected ACKR3-Venus throughout brain vasculature on CD31 and vimentin positive vasculature structures (Fig. 8). This is in line with other observations of ACKR3 immunoreactivity in brain vasculature [30, 69]. ACKR3 likely plays important roles in angiogenesis and maintenance of brain vasculature as well as the blood brain barrier (BBB). ACKR3 at the BBB has recently been shown to play a protective in neuroimmunological axis, neurodegeneration and memory loss [26]. Neuroinflammation caused by repeated exposures to stressful stimuli have been shown to contribute to depression [70].

Finally, anti-depressants, including tianeptine a partial mu opioid receptor agonist [71] and fluoxetine, have been shown to normalize ACKR3 transcript and protein levels when precipitated by prenatal stress in frontal cortex of rats [72] suggesting that ACKR3 modulation may be therapeutic in psychiatric disease. Notably, ACKR3 was previously reported to be responsive opioid peptides in adrenocortical cells contributing to anxiolytic behavior in mice [73]. Recently, ACKR3, although not activated by prescription opioids, was demonstrated to broadly scavenge opioid peptides [22]. These findings suggest that ACKR3 is a potential therapeutic target for modulating depression and possibly pain, perhaps by inhibiting ACKR3, excess endogenous opioid peptides can remain in circulation and help to reduce neuroinflammation or alleviate pain.

In summary, we have generated *Ackr3-Venus* knock-in mice with a traceable ACKR3 receptor. The receptor is directly visible, and the signal can easily be amplified using antibodies directed against the tag. These animals will be valuable for future interrogations into the subcellular distribution of ACKR3 either in living acute sections or in ex-vivo primary cells. Tissue distribution of ACKR3-Venus aligns with previous reports and in HEK-293 cells, we observed normal signaling responses. This mouse should therefore be a useful tool to the research community for interrogations about ACKR3 and its critical roles in angiogenesis, inflammation, cancer and psychiatric diseases like depression and addiction.

## Material and methods

### Reagents

The compounds Met-Enkephalin (Genscript), AMD3100, TC14012 and VUF22207 (Tocris Biosciences) and CXCL12 (R&D systems). The Coelenterazine H and 400a were purchased from Nanolight.

### Plasmids

The mouse coding sequence of mouse βArrestin2 and mouse GRK2 were subcloned into pcDNA3.1 using Gibson assembly (NEB) with the following primers integrating the restriction site HindIII: mouse βArrestin2 fwd 5′-gtttaaacttaagcttcaattgccgccaccatgggagaaaaacccgggaccagggtcttca-3′, mouse βArrestin2 rvs 5′- actagtggatccgagctcggtaccaagcttgcagaactggtcatcacagtcatcatcc-3′, mouse GRK2 fwd 5′-gtttaaacttaagcttcaattgccgccaccatgcagaagtatctggaggaccgaggagaa-3′ and mouse GRK2 rvs 5′-actagtggatccgagctcggtaccaagcttgaggccgttggcactgccacgctggatcag-3′.

The mouse coding sequence of ACKR3 was PCR amplified and subcloned into pIRES (Invitrogen) and pIRES-Venus vector previously described [74] using Gibson assembly with the following primers integrating the restriction site BamHI: mACKR3 fwd 5′-tatctgcggcctagctagccaccaggatccgccaccatggatctgcatctcttcgactac-3′ for both constructions and mACKR3 rvs 5′-ccagcacactgggcccggtggcgatggatcctttggtgctctgctccaaggcagagtactc-3′ for pIRES-ACKR3 and 5′-gacagcgaattaattccagcacactgggcccttacttatacagctcgtccatgccgagagt-3′, for pIRES-ACKR3-Venus.

The Flag tagged versions of ACKR3 and ACKR3-Venus were made by subcloning the coding sequence of ACKR3 or ACKR3-Venus from the pIRES-ACKR3 or ACKR3-Venus constructs. Using in-fusion cloning (Takara), the coding sequences were subcloned into pCAGGSSF-G418 vector, a modified version of pGCGFP-G418 (addgene #31264) with the GFP coding sequence removed, at the NotI site. For both constructs ACKR3 fwd 5′-gctggagcagcggccatggatgtgcacttgtttgactatgc-3′ was used. For pCAGGSSF-ACKR3 rvs 5′-tctagagtcgcggcctcacttggtgttctgttccaggg-3′. For pCAGGSSF-ACKR3-Venus rvs 5′- tctagagtcgcggccttacttatacagctcgtccatgcc-3′.

The mouse βArrestin2-RlucII and rGFP-CAAX BRET biosensor constructs were generated as previously described [34].

### Cell culture and transfections

HEK-293 cells were maintained in Dulbecco’s modified Eagle's medium (DMEM) supplemented with 10% new calf serum and 100 U/mL penicillin/streptomycin (Wisent) in a 37 °C humidified incubator with a 5% CO_2_ atmosphere. Two days prior to experiments, cells were trypsinized (Wisent) and 35,000 cells were transfected with 100 ng of total DNA containing the appropriate expression vectors/biosensors. The total quantity of DNA was completed at 100 ng with salmon sperm DNA (Invitrogen). Transfection was performed using the transfecting agent polyethylenimine 25 kD linear (PEI; Polysciences) at a ratio of 3:1 PEI/DNA. Cells were then immediately plated onto poly-ornithine (Sigma-Aldrich) coated 96-well white culture plates (PerkinElmer). For Flag-ACKR3 experiments, 1 µL of Lipofectamine 2000 (Thermo Fisher) was used to transfect 400 ng of DNA in a 24 well format.

### Staining surface flag-ACKR3 and flag-ACKR3-Venus

HEK-293 cells were plated on 12 mm coverslips (NeuVitro) at 8 × 10^4^ cells per well the day before transfection. 24 h after transfection, M1-Alexa 647 Flag antibody was added to the cells at 1:2000 at 37 °C for 15 min. Cells were washed in cold PBS (Thermo Fisher), fixed with 4% paraformaldehyde or PFA/PBS for 10 min and permeabilized in PBS-T (0.1% Triton X-100, Sigma) for 10 min. Cells were blocked with 3% BSA (Sigma)/0.2% Triton X-100/PBS, washed in PBS-T 3 times, and stained with DAPI (1:10,000, Thermo Fisher) in PBS FOR 10 min followed by a PBS wash and mounting the coverslips onto slides (Thermo Fisher) in Prolong Diamond (Molecular Probes). Confocal images were acquired on a Nikon CSU-W1 spinning disk confocal microscope with a Plan-Apochromat 60×/1.27 Oil lens. Detection for DAPI was with excitation at 405 nm and emission at 450/50 m and for Flag, excitation at 647 nm with emission filters 620/60 m. Experiments were repeated 3 times and 10 cells per condition were imaged in each experiment.

### Live-cell imaging of ACKR3-Venus in HEK-293 cells

For time-dependent agonist induced internalization, Fig. 1F, HEK-293 cells were seeded into poly-D-lysine-coated 35 mm glass bottom culture dishes (P35GC-1.5–14-C, Mattek corp) at the density of 2 × 10^5^ cells/dish. Cells were maintained in DMEM supplemented with 10% newborn calf serum at 37 °C 5% CO_2_. 24 h after the seeding, cells were transfected with 50–100 ng of CXCR7-Venus using X-tremeGENE 9 DNA transfecting reagent (Roche) according to the manufacturer protocol. 48 h after the transfection, cells were washed twice with modified Hank’s balanced salt solution (HBSS; 138 mM NaCl, 5.3 mM KCl, 0.44 mM KH2PO4, 0.33 mM Na_2_HPO_4_, 1 mM CaCl_2_, 1 mM MgCl_2_, 10 mM HEPES pH 7.4), incubated at room temperature in HBSS supplemented with 0.1% bovine serum albumin, and stimulated with 300 nM CXCL12 (CLY100-202, Cedarlane) just before the fluorescence imaging. Confocal microscopy was carried out using LSM880 (Carl Zeiss) equipped with 514 nm argon laser line and Plan-Apochromat 63×/1.4 lens. Detection wavelength was 528–600 nm and images were obtained with 0.9 AU pinhole aperture.

### ACKR3 scavenging experiments

HEK-293 cells were transfected (Nucleofection according to manufacturer’s instruction) with either wild type mouse ACKR3 or ACKR3-Venus. Cells were plated on Mattek glass bottom dishes. 24 h later, cells were stimulated with 50 nM CXCL11-12AF647 for 20 min at 37 °C. Reactions were terminated by fixing with 4% PFA in PBS for 10 min at RT. Cells were permeabilized for 10 min with PBS/0.1% Triton X-100. Supernatant was removed and cells embedded in the presence of DAPI.

### BRET measurement

Forty-eight hours post-transfection, cells were washed once with phosphate-buffered saline (PBS) then incubated 1 h at 37 °C in Tyrode buffer (137 mM NaCI, 0.9 mM KCI, 1 mM MgCI2, 1 1.9 mM NaHCO3, 3.6 mM NaH2PO4, 25 mM HEPES, 5.5 mM Glucose and 1 mM CaCI2, pH 7.4). Cells were then treated with the ligands and prior to measurement treated with the substrate (15 min with 2.5 µM Coelantarazine H for BRET^1^ assays, and 5 min with 2.5 µM Coelantarazine 400a for BRET^2^ and ebBRET assays). Bioluminescence resonance energy transfer (BRET) was then measured between RlucII (BRET energy donor) and either Venus (BRET^1^ energy acceptor) or GFP_10_ (BRET^2^) or rGFP (enhanced bystander ebBRET acceptor)–tagged proteins. BRET values were read for 1 s per well using a Tristar^®^LB942 Multimode Microplate Reader (Berthold Technologies) and a Mithras™ LB940 Multimode Microplate Reader (Berthold Technologies) for kinetics and concentration–response curves, respectively. BRET^1^ and ebBRET values were obtained by calculating the ratio of the light emitted by the energy acceptor over the light emitted by the energy donor (donor 480 ± 20 nm/acceptor 530 ± 20 nm for BRET^1^ and 410 ± 70 nm/acceptor 515 ± 20 nm for ebBRET). Data were collected using the MicroWin 2000 software (Berthold Technologies). They were then fitted and analyzed in GraphPad Prism (v7.0, GraphPad Software, Inc).

### Animals

The *Ackr3-Venus* mouse line was established by PHENOMIN at the MCI/ICS (Mouse Clinical Institute—Institut Clinique de la Souris-, Illkirch, France; http://www.phenomin.fr/en-us/). The targeting vector was constructed as follows (Fig. 3A). A 3.5 kb fragment encompassing part of *Ackr3* intron 1 was amplified by PCR (from RP23-338M8 BAC containing *Ackr3*) and subcloned in an ICS proprietary vector. This ICS vector bares a floxed Neomycin resistance cassette associated with a Cre autoexcision transgene that will allow the excision of the whole cassette in the chimera’s male germ line. Three fragments of, (1) 758 bp for the linker-Venus fragment, (2) 1410 bp for the partial intron 1-exon2 fragment and (3) 3560 bp for the end of exon 1 (3′UTR) and genomic region were amplified by PCR and subcloned by SLIC cloning in step1 plasmid to generate the final targeting construct. The linearized construct was electroporated in C57BL/6N mouse embryonic stem (ES) cells (ICS proprietary line). After G418 selection, targeted clones were identified by long-range PCR and further confirmed by Southern blot with an internal (Neo) probe and a 5′ external probe. Two positive ES clones were validated by karyotype spreading and microinjected into BALB/C blastocysts. Resulting male chimeras were bred with wild type C57BL/6N females. Germline transmission with the direct excision of the selection cassette was achieved in the first litter.

*Ackr3*^*Venus/WT*^ mice were intercrossed to generate *Ackr3*^*Venus/Venus*^ mice, which are fertile and develop normally. *Ackr3*^*WT/WT*^, *Ackr3*^*Venus/WT*^ and *Ackr3*^*Venus/Venus*^ mice aged 8–16 weeks were bred in-house (Neurophenotyping Center, McGill University/Douglas Hospital Research Institute, Montreal, Canada) on a C57Bl/6N (Charles River Laboratories) background. Animals were group-housed and maintained on a 12-h light/dark cycle (lights on at 8:00 AM) at a controlled temperature (22 °C ± 1 °C). Food and water were available ad libitum throughout all experiments, unless otherwise stated. All experimental procedures were performed in accordance with the guidelines of the Canadian Council of Animal Care, and all animal procedures were approved by the McGill University/Douglas Hospital Animal Care Committee. All animals were routinely genotyped at weaning and after experimental endpoints using the following genotyping primers: Forward 5′-GCTGTGGCCAGGGCTCTACTAT-3′ Reverse 5′-CTTGAGGAGAGCGACCAAGTGA-3′ which produced the following sized bands for each genotype: 418 bp—*Ackr3*^*WT/WT*^, 339 bp—*Ackr3*^*Venus/Venus*^ mice, both bands are visible for *Ackr3*^*Venus/WT*^.

### RNA sample preparation and isolation

RNA was collected using freshly dissected brains on ice, brains were severed along the midline and hemispheres were immediately frozen on dry ice and stored at – 80 °C until use. TRIzol (Invitrogen) reagent was added directly into the collected sample tubes. Mechanical dissociation was applied to yield a homogenate and tissue extraction was done according to the manufacture’s protocol. The precipitate carrier, Dr. Gentle (Takara), was added to visualize RNA pellets. RNase-and DNase- free water was used to resuspend the Total RNA (ThermoFisher). Purity and concentration of total RNA was evaluated on a NanoDrop ND2000 (ThermoFisher).

### RT-qPCR

RT-qPCR was performed as described previously [43]. Briefly, 400 ng of RNA was reverse transcribed using the M-MLV Reverse Transcriptase Kit (Invitrogen) according to the manufacturer’s instructions. cDNA was diluted 20 times in RNase-and DNase- free water. 2 μl of cDNA was combined with forward and reverse primers at final concentration of 0.5 μM each primer and 5 μl of LightCycler 480 SYBR I Green Master Mix (Roche). cDNA samples were loaded in triplicate with 10 μl final volume adjusted with RNase-and DNase- free water. Samples were run in 384-well white polypropylene plates (Roche) for 45 cycles of amplification on the LightCycler 480 II Real-Time PCR System (Roche). A no template control (NTC) reaction, with just water, was included to check for non-specific amplification. The housekeeping gene, *B2m,* average was subtracted from the average of the triplicate CT values for each sample. Relative fold changes were calculated by the comparative CT method (2^−ΔΔCT^) [75] and multiplied by 100 to show the data in percent.

### Table of RT-qPCR primers

**Table:** 

Gene	Forward	Reverse
*Venus*	CACATGAAGCAGCACGACTT	CATTGTGGGCGTTGTAGTTG
Ackr3	AGGAAGCCCTGAGGTCACTT	CAATGCAGTCGCTGCTGTTAC
mGluR5	TGGCAAGTCATCATCCGCTG	TTTCCATTGGAGCTTAGGGTTTCC
*BetaActin*	GACGGCCAGGTCATCACTAT	CCACCGATCCACACAGAGTA
*B2m*	TGGTGCTTGTCTCACTGACC	GTATGTTCGGCTTCCCATTC

### Immunohistochemistry

#### Immunohistochemistry on *Ackr3-venus* KI mouse peripheral organs

Mice aged 8–10 weeks were euthanized in a CO_2_ chamber. Internal organs were removed and fixed in formalin 10% (FisherScientific) for 24 h. The fixed organs were then embedded in paraffin, transversally sliced (4 µm), deposited onto slides (ThermoFisher) and immunostaining was carried out directly on the slide using the automated Bond RX staining platform (Leica Biosystems). Sections were deparaffinised inside immunostainer. Antigen recovery was conducted using Heat-Induced Epitope Retrieval with Leica Biosystems proprietary Epitope Retrieval ER1, using a low pH buffer for 20 mn. Sections were then incubated with 150 µL of mouse GFP antibody (ab290, Abcam; 1/10000) for 30 mn at room temperature. Detection of specific signal was acquired by using Bond Polymer DAB Refine kit (#DS9800, Leica Biosystems) with a 15 mn incubation time for polymer anti-rabbit reagent. Slides were counterstained automatically with hematoxylin and eosin included in the detection system. Stained slides were coverslipped and scanned using the Hamamatsu's NanoZoomer^®^ Digital Pathology system 2HT (Hamamatsu).

#### Immunohistochemistry on *Ackr3-Venus* KI mouse brain

Immunohistochemistry was carried out on coronal brain sections from animals as previously described [43]. Briefly, before dissection animals were anesthetized with i.p. injections of 100 μl/100 g of a cocktail containing Ketamine/Xylazine/Acépromazine. Male and female mice were then intracardially perfused with 10 ml of 1 × PBS pH 7.4 (ThermoFisher) followed by 50 ml of 4% PFA (Cedarlane) in 1 × PBS pH 7.4. Brains were extracted from skulls using surgical tools (Dumont) and immersed in 10 ml 4% PFA/1 × PBS overnight. The next day, brains were transferred into 10 ml of 30% sucrose (ThermoFisher) 1 × PBS pH 7.4 to cryoprotect at 4 °C. Brains were embedded in O.C.T. (Sakura Finetek) and stored at – 80 °C. Brains were sectioned at 30 μm on a cryostat (Leica) set at – 20 °C. Brain sections were kept free floating in a 24-well dish containing 1 × PBS pH 7.4. On the day of staining, selected brain section (180 μm apart) were permeabilized in PBS-T (1 × PBS pH 7.4, 0.1% Triton X-100) followed by incubation in blocking buffer for one-hour (1 × PBS, normal goat serum (NGS; Sigma-Aldrich), 0.2% Triton X-100). Primary antibodies (see table below) were incubated overnight at 4 °C. The next day, tissue was washed 3 times in PBS-T. Secondary antibody, Alexa Fluor 488, Alexa Fluor 594 or Alexa Fluor 647 (1:2000, ThermoFisher), was incubated with tissue sections for 2 h at room temperature with gentle agitation. Following washes, brain sections were mounted onto slides and coverslips (ThermoFisher) were sealed with Moviol (Sigma-Aldrich) with or without 4′,6-diamidino-2 phenylindole (DAPI; ThermoFisher).

**Table:** 

Antibody	Concentration	Catalog#	Manufacturer
CD31(PECAM-1)	1:50	Ab28364	Abcam
DCX	1:2000	AB2253	Millipore Sigma
GABA	1:1000	A2052	Sigma
GFAP	1:2000	ab4674	Abcam
NeuN	1:2000	24307S	Cell Signaling Technology
NG2	1:200	AB5320	Millipore Sigma
Venus (GFP)	1:2000	A111222	ThermoFisher
Venus (GFP)	1:2000	NB100-1614	Novus Bio
Vimentin	1:100	5741S	Cell Signaling Technology

### Microscopy

For epifluorescence images captured on an Olympus IX73 microscope (Fig. 5A and Additional file 1: Figure S4), filter cubes were used as indicated (DAPI em. 455 nm, intrinsic Venus em. 527 nm, Alexa Fluor 594 Cy3 em. 565 nm). For the slide scanner VS120 (Olympus Corporation) images (Fig. 6), whole slides were scanned with a 10 × objective (red, was detected using TRITC filter Ex. 535 ± 36 nm, Em. 590 ± 34 nm). Images were analyzed using NDP viewer (Hamamatsu) software and exported images were obtained using FIJI ImageJ [76]. Confocal images (Figs. 5B, 7, 8, Additional file 1: Figure S5) were acquired on a laser scanning Olympus FV1200 (Olympus Corporation) Original single plane images were acquired using 20 × objective lens (Alexa Fluor 488 Ex. 488/Em. 520, Venus ex. 518/ em. 543, Alexa Fluor 594 Ex. 543/Em. 618, Alexa Fluor 647 Ex. 635/Em. 647).

### Mapping ACKR3-venus

To score the expression of ACKR3-Venus throughout the mouse brain, 30 µm coronal sections with 180 µm between sections were imaged on an epifluorescent slide scanner. Images were scored across 654 brain regions according to the mouse brain atlas [77]. *Ackr3*^*WT/WT*^ (n = 3) and *Ackr3*^*Venus/Venus*^ (n = 4) were examined (Table 3, Fig. 6 and Additional file 1: Figure S4). All sections were scanned on the slide scanner with a 10 × objective. The level of ACKR3-Venus expression amplified by a Venus antibody red, was detected using TRITC filter Ex. 535 ± 36 nm, Em. 590 ± 34 nm) was scored by comparing the fluorescence signal between background level (*Ackr3*^*WT/WT*^*)* and corresponding *Ackr3*^*Venus/Venus*^ brain areas. This analysis was qualitative, and no statistical analysis has been done. A scale of four levels of fluorescence was used to determine ACKR3-Venus expression at each region [very low (+), low (+ +), moderate (+ + +), high (+ + + +)]. Data for all animals/brain area was pooled to generate a final score. If a region obtained a score which differed by a degree of 2 between animals the region was not reported. Thus, only the brain regions with scores of the highest confidence were selected to include in the table. We then cross-compared the brain regions between the mouse brain atlas [77] with the Allen Brain Interactive Atlas Viewer (http://atlas.brain-map.org/) and consolidated small sub-groups into main brain regions according to the latter atlas, as indicated in Table 3.

## Supplementary Information

**Additional file 1: Figure S1.** G protein activation profile. **A**–**C** HEK-293 cells were co-transfected with the indicated receptor (hDOR, mACKR3 or mACKR3-Venus), Gβ1, hGγ3-RlucII (BRET2 sensor donor), hGRK2-GFP10 (BRET2 sensor acceptor) and the indicated human (h) or mouse (m) Gα subunits. Mock condition is with cells not co-transfected with a Gα subunit encoding cDNA, thus detecting receptor-mediated activation of endogenous G proteins. **A** G protein activation profile of the human Delta opioid receptor (hDOR) following a 10 min stimulation with Met-Enkephalin (30 µM). **B**, **C** G protein activation profile of the untagged (ACKR3) or Venus-tagged (ACKR3-Venus) mouse ACKR3 receptor following a 10 min stimulation with CXCL12 (1 µM). Data are expressed as the mean ± SEM of 3 independent experiments. **D** HEK-293 cells were transiently transfected with ACKR3 or ACKR3-Venus and stimulated with 50 nM CXCL11-12AF647 for 20 min at 37 °C and scavenging was assessed by confocal microscopy. Nuclei were stained with DAPI. Cells not expressing ACKR3 or ACKR3-Venus (on the right, DAPI positive) did not take up CXCL11-12. **Figure S2.** H&E staining of ACKR3-Venus expression in peripheral mouse organs. Representative images of amplified ACKR3-Venus expression from eight peripheral tissues prepared from adult (P56 to P72) female and male mice. Organs from Ackr3Venus/Venus mice were sectioned transversely (4µm), and the expression of Venus was revealed with a mouse Venus (GFP) antibody (brown) and counterstained with hematoxylin labeling of cell nuclei (blue) and eosin labelling of cytoplasm (pink). Shown are representative images of organs where ACKR3-Venus is observed **A** heart (male) **B** stomach (female) **C** intestine (male) **D** kidney (female) **E** Thymus (female) **F** colon (female) **G** lung (male) **H** Fat (male). Arrowheads are located at regions with ACKR3-Venus expression. A yellow box on the inset indicates where the higher magnification image was taken. Organ sections were imaged on the Hamamatsu’s NanoZoomer^®^ Digital Pathology system 2HT with 80× (large image) and 20× (inset) objectives. Scale bar is 25µm. **Figure S3.** ACKR3-Venus expression in spleen. Representative images of amplified ACKR3-Venus expression from adult (P56 to P72) female and male mice. Organs from Ackr3Venus/Venus mice were sectioned transversely (4 µm) and the expression of Venus was revealed with a mouse Venus (GFP) antibody. The consecutive slices were incubated with hematoxylin and eosin (H&E) staining to reveal the histological structure of the spleen. Shown are representative images where ACKR3-Venus is observed, **A** Red pulp **B** Peripheral region of white pulp **C** Central region of white pulp **D** Marginal zone **E** Red pulp (H&E) **F** Peripheral region of white pulp (H&E) **G** Central region of white pulp (H&E) **H** Marginal zone (H&E). Arrowheads are located at regions with ACKR3-Venus expression. A red or yellow box on the inset indicates where the higher magnification image was taken. Organ sections were imaged on the Hamamatsu’s NanoZoomer^®^ Digital Pathology system 2HT with 80× (large image) and 20× (inset) objectives. Scale bar is 25µm. **Figure S4.** ACKR3-Venus expression in female peripheral organs. Representative images of amplified ACKR3-Venus expression from twelve peripheral tissues prepared from adult (P56 to P72) female mice. In addition to the eight organs which show considerable ACKR3-Venus expression (see Figure 5), shown here are all 12 organs side-by-side with the comparable control Ackr3WT/WT samples. Organs from Ackr3Venus/Venus mice were sectioned transversely (4 µm), and the expression of Venus was revealed with a mouse Venus (GFP) antibody (brown) and counterstained with hematoxylin labeling of cell nuclei (blue). Shown are representative images of the following organs **A** fat **B** stomach **C** intestine **D** colon **E** liver **F** lung **G** pancreas **H** heart **I** thymus **J** kidney **K** uterine horns **L**–**N** spleen **L** red pulp **M** marginal zone **N** white pulp. Expression in Ackr3Venus/Venus (n = 3) were compared to any background staining observed in Ackr3WT/WT (n = 3). A red box on the inset indicates where the higher magnification image was taken. Organ sections were imaged on the Hamamatsu’s NanoZoomer^®^ Digital Pathology system 2HT with 80× (large image) 20× (inset) objectives. Scale bar is 25 µm. **Figure S5.** H&E staining with ACKR3-Venus expression in female peripheral organs. Representative images of amplified ACKR3-Venus expression from twelve peripheral tissues prepared from adult (P56 to P72) female mice. In addition to the eight organs which show considerable ACKR3-Venus expression (see Figure 4 and S3), shown here are all 12 organs side-by-side with the comparable control Ackr3WT/WT samples. Organs from Ackr3Venus/Venus mice were sectioned transversely (4 µm), and the expression of Venus was revealed with a mouse Venus (GFP) antibody (brown) and counterstained with hematoxylin and eosin (H&E) staining to reveal the histological structure. Shown are representative images of the following organs **A** fat **B** stomach **C** intestine **D** colon **E** liver **F** lung **G** pancreas **H** heart **I** thymus **J** kidney **K** uterine horns **L**–**N** spleen **L** red pulp **M** marginal zone **N** white pulp. Expression in Ackr3Venus/Venus (n = 3) were compared to any background staining observed in Ackr3WT/WT (n = 3). A yellow box on the inset indicates where the higher magnification image was taken. Organ sections were imaged on the Hamamatsu’s NanoZoomer^®^ Digital Pathology system 2HT with 80× (large image) 20× (inset) objectives. Scale bar is 25 µm. **Figure S6.** ACKR3-Venus expression in male peripheral organs. Representative images of amplified ACKR3-Venus expression from thirteen peripheral tissues prepared from adult (P56 to P72) male mice. In addition to the eight tissues which show considerable ACKR3-Venus expression (see Figure 4 and S3), shown here are all 13 organs side-by-side with the comparable control Ackr3WT/WT samples. Organs from Ackr3Venus/Venus mice were sectioned transversely (4 µm), and the expression of Venus was detected with a mouse Venus (GFP) antibody (brown) and counterstained with hematoxylin labeling of cell nuclei (blue). Shown are representative images of the following organs **A** fat **B** stomach **C** intestine **D** colon **E** liver **F** lung **G** pancreas **H** heart **I** thymus **J** kidney **K** testis **L** seminal vesicles **M**–**O** spleen **M** red pulp **N** marginal zone **O** white pulp. Expression in Ackr3Venus/Venus (n = 3) were compared to any background staining observed in Ackr3WT/WT (n = 3). A red box on the inset indicates where the higher magnification image was taken. Organ sections were imaged on the Hamamatsu’s NanoZoomer^®^ Digital Pathology system 2HT with 80× (large image) 20× (inset) objective. Scale bar is 100µm. **Figure S7.** H&E staining with ACKR3-Venus expression in male peripheral organs. Representative images of amplified ACKR3-Venus expression from twelve peripheral tissues prepared from adult (P56 to P72) male mice. In addition to the eight organs which show considerable ACKR3-Venus expression (see Figure 4 and S3), shown here are all 13 organs side-by-side with the comparable control Ackr3WT/WT samples. Organs from Ackr3Venus/Venus mice were sectioned transversely (4µm), and the expression of Venus was revealed with a mouse Venus (GFP) antibody (brown) and counterstained with hematoxylin and eosin (H&E) staining to reveal the histological structure. Shown are representative images of the following organs **A** fat **B** stomach **C** intestine **D** colon **E** liver **F** lung **G** pancreas **H** heart **I** thymus **J** kidney **K** testis **L** seminal vesicles **M**–**O** spleen **M** red pulp **N** marginal zone **O** white pulp. Expression in Ackr3Venus/Venus (n = 3) were compared to any background staining observed in Ackr3WT/WT (n = 3). A yellow box on the inset indicates where the higher magnification image was taken. Organ sections were imaged on the Hamamatsu’s NanoZoomer^®^ Digital Pathology system 2HT with 80× (large image) 20× (inset) objectives. Scale bar is 25 µm. **Figure S8.** Intrinsic and amplified cellular expression of ACKR3-Venus in the adult brain of Ackr3WT/WT mice. To check for non-specific tissue signals generated by naturally occurring autofluorescence or non-specific interactions between antibodies, we immunostained Ackr3WT/WT brain sections (n = 4) and observed negligible background signals. Images were taken on Olympus IX73 epifluorescent microscope, 10× objective. Scale bar is 200 µm. **Figure S9.** Oligodendrocyte progenitor cell staining in Ackr3-Venus adult mouse brain. To examine oligodendrocyte progenitor cells, brain sections from Ackr3Venus/Venus mice were stained with anti-NG2 (cyan), a marker of oligodendrocytes progenitor cells and anti-Venus (red). Shown regions include **A** the olfactory bulb (OB), **B** orbital frontal cortex (OFC) and **C** Hippocampus (HPF). Amplified Venus and NG2 expression in brain sections were imaged on an Olympus FV1200 laser scanning confocal microscope, 20× objective. White boxes (left column) correspond to the 5× magnified images. Insets contain mouse brain reference atlas images from Allen Brain Atlas, with the region locations outlined by black boxes. Scale bar of 20× image is 100 µm (1st column) and 20 µm (2nd–3rd columns).

## Data Availability

The datasets during and/or analyzed during the current study available from the corresponding author on reasonable request.
