# Supplementary material for: Ackr3-Venus knock-in mouse lights up brain vasculature
Source: Mol Brain. 2021 Sep 28;14:151. doi: 10.1186/s13041-021-00862-y (PMC8477500; doi:10.1186/s13041-021-00862-y)
Supplement: Supplementary file 1 — Additional file 1: Figure S1. G protein activation profile. A–C HEK-293 cells were co-transfected with the indicated receptor (hDOR, mACKR3 or mACKR3-Venus), Gβ1, hGγ3-RlucII (BRET2 sensor donor), hGRK2-GFP10 (BRET2 sensor acceptor) and the indicated human (h) or mouse (m) Gα subunits. Mock condition is with cells not co-transfected with a Gα subunit encoding cDNA, thus detecting receptor-mediated activation of endogenous G proteins. A G protein activation profile of the human Delta opioid receptor (hDOR) following a 10 min stimulation with Met-Enkephalin (30 µM). B, C G protein activation profile of the untagged (ACKR3) or Venus-tagged (ACKR3-Venus) mouse ACKR3 receptor following a 10 min stimulation with CXCL12 (1 µM). Data are expressed as the mean ± SEM of 3 independent experiments. D HEK-293 cells were transiently transfected with ACKR3 or ACKR3-Venus and stimulated with 50 nM CXCL11-12AF647 for 20 min at 37 °C and scavenging was assessed by confocal microscopy. Nuclei were stained with DAPI. Cells not expressing ACKR3 or ACKR3-Venus (on the right, DAPI positive) did not take up CXCL11-12. Figure S2. H&E staining of ACKR3-Venus expression in peripheral mouse organs. Representative images of amplified ACKR3-Venus expression from eight peripheral tissues prepared from adult (P56 to P72) female and male mice. Organs from Ackr3Venus/Venus mice were sectioned transversely (4µm), and the expression of Venus was revealed with a mouse Venus (GFP) antibody (brown) and counterstained with hematoxylin labeling of cell nuclei (blue) and eosin labelling of cytoplasm (pink). Shown are representative images of organs where ACKR3-Venus is observed A heart (male) B stomach (female) C intestine (male) D kidney (female) E Thymus (female) F colon (female) G lung (male) H Fat (male). Arrowheads are located at regions with ACKR3-Venus expression. A yellow box on the inset indicates where the higher magnification image was taken. Organ sections were imaged on the Hamamatsu’s NanoZoom [file 13041_2021_862_MOESM1_ESM.pdf]

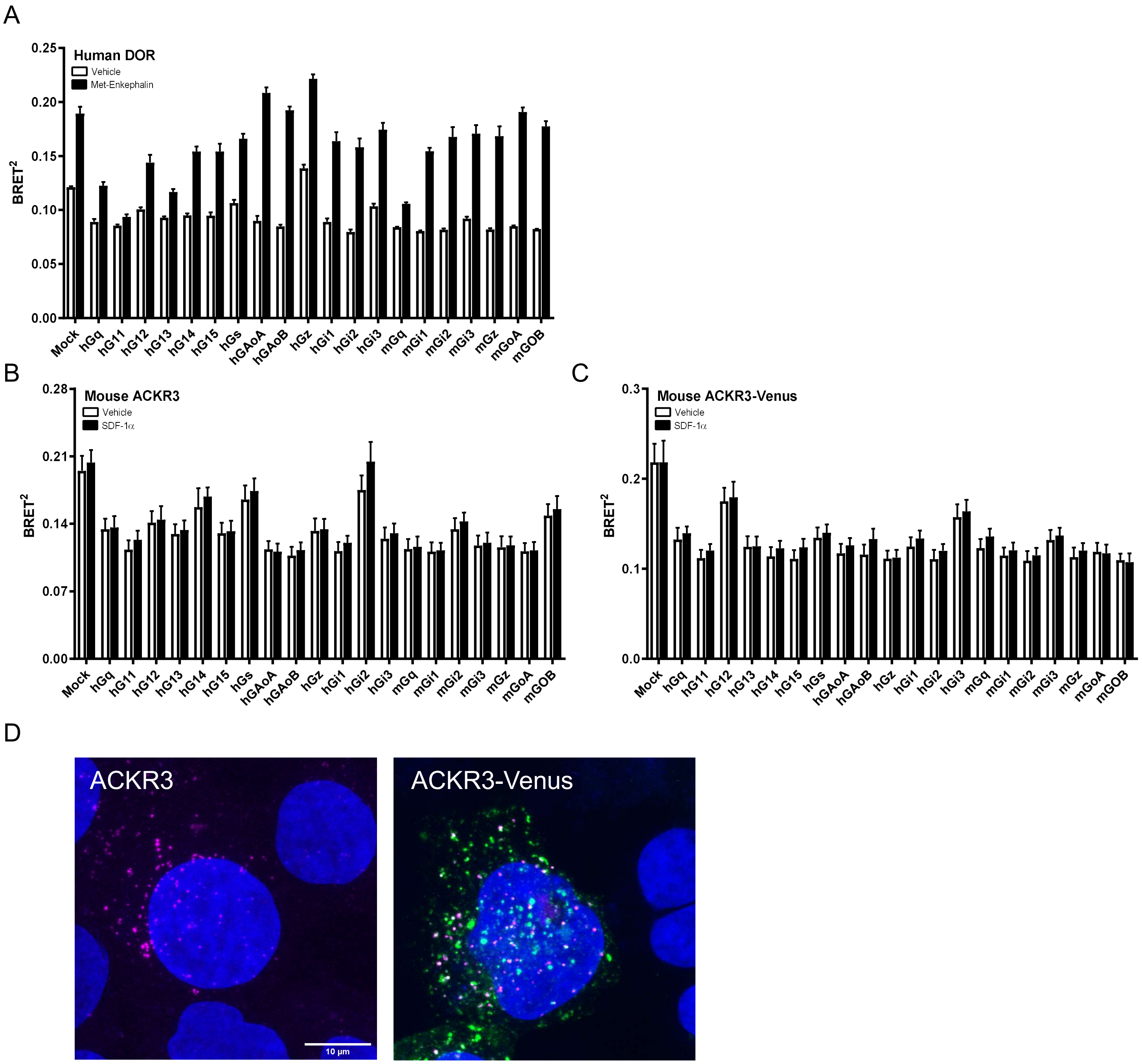

Supplemental Figure S1.

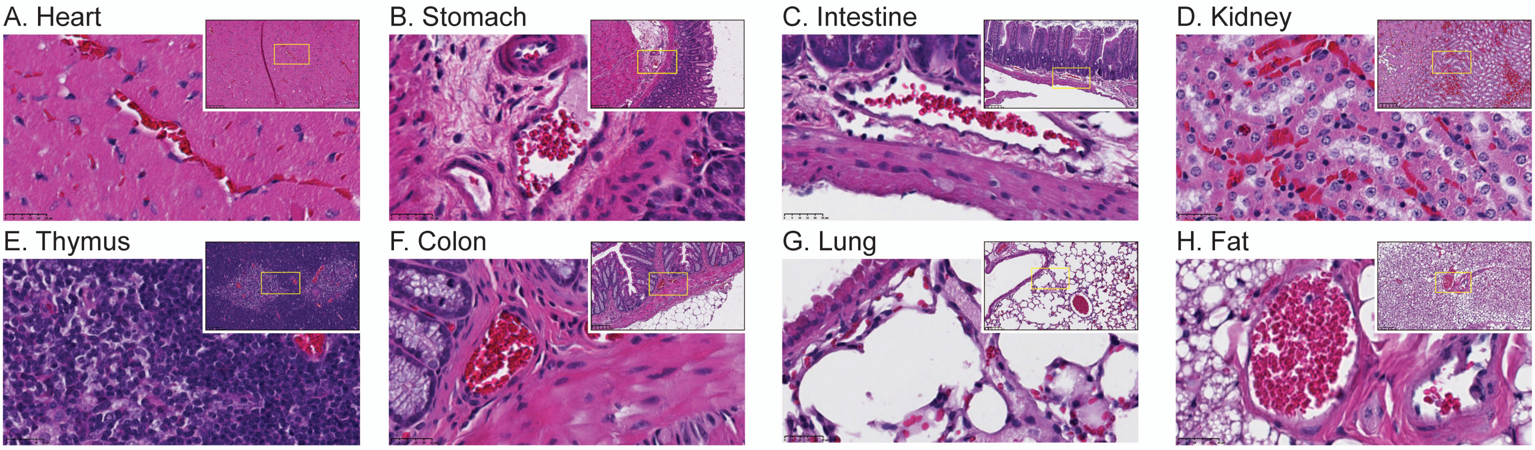

Supplemental Figure S2.

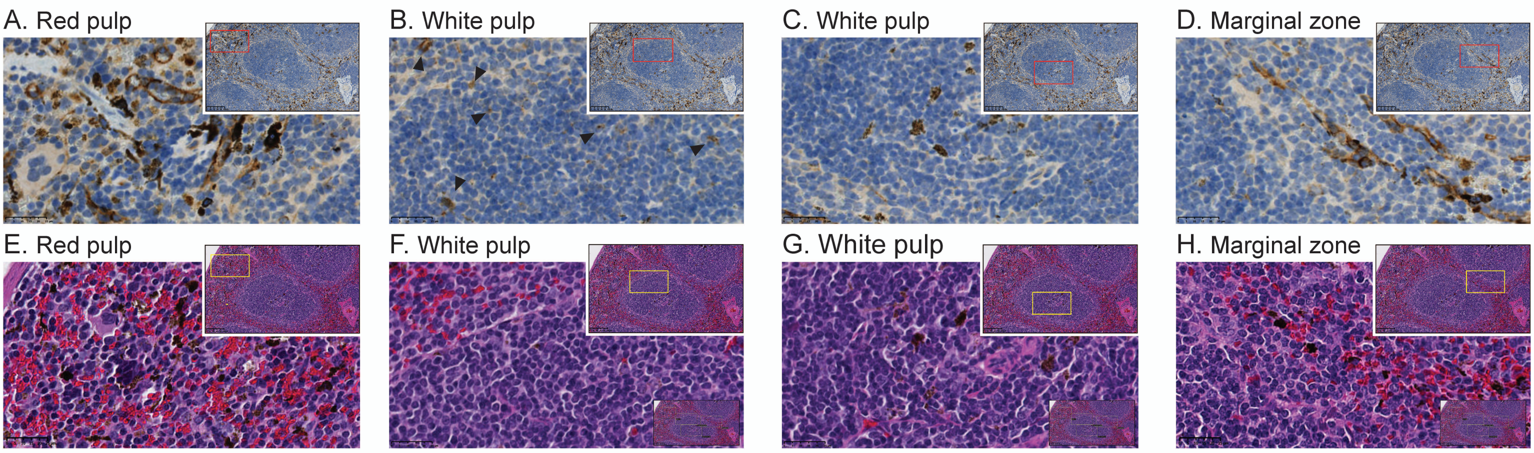

Supplemental Figure S3.

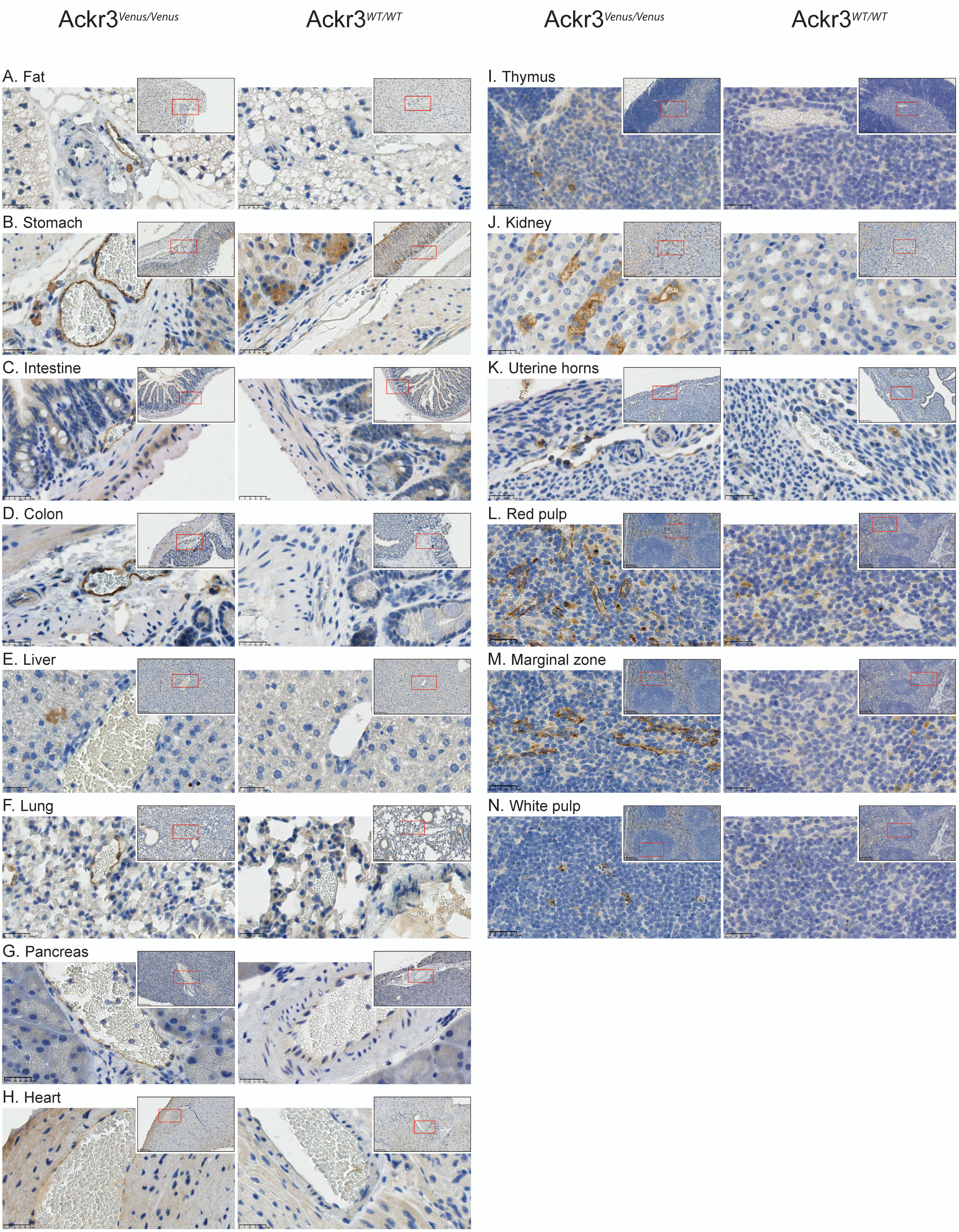

Supplemental Figure S4.

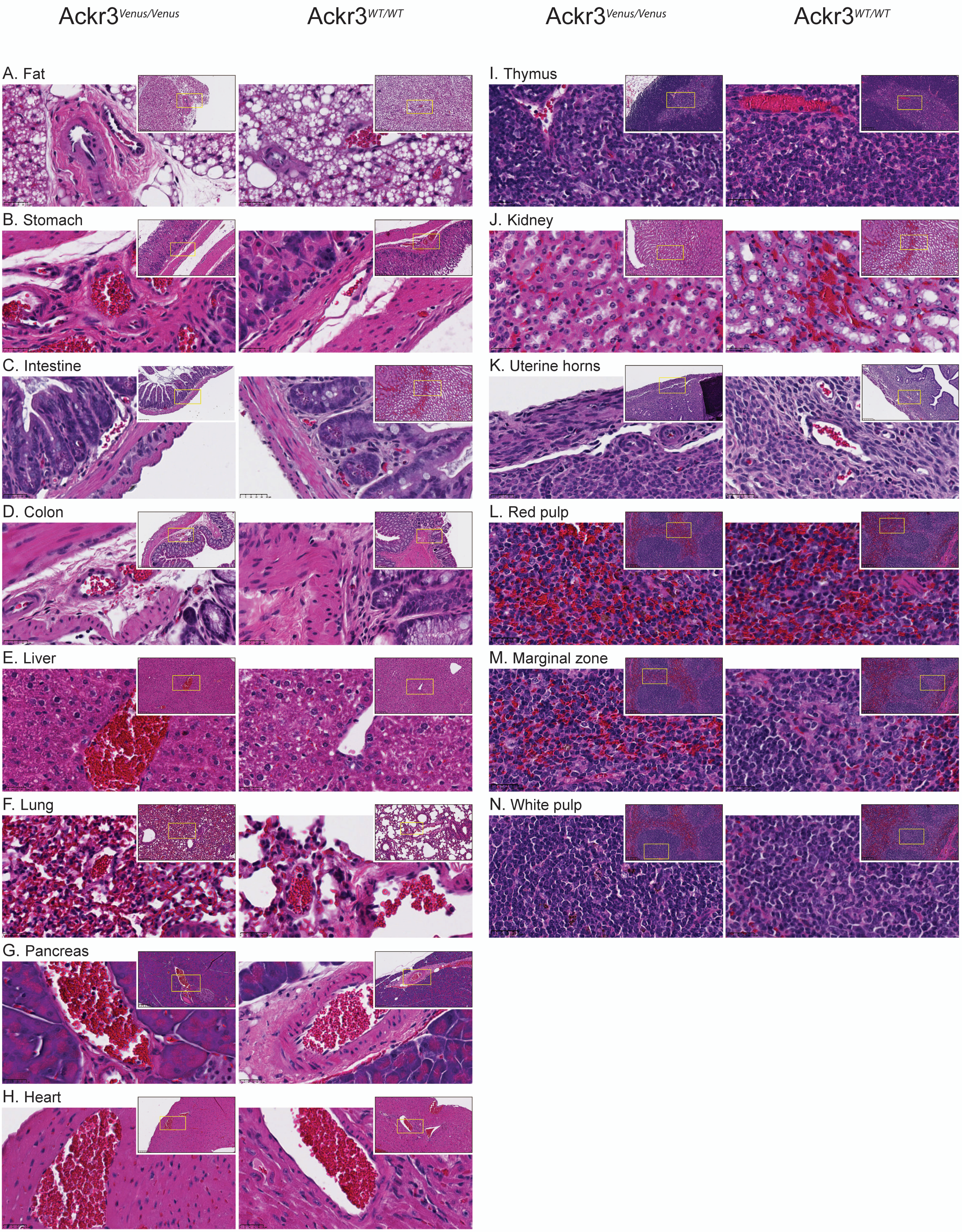

Supplemental Figure S5.

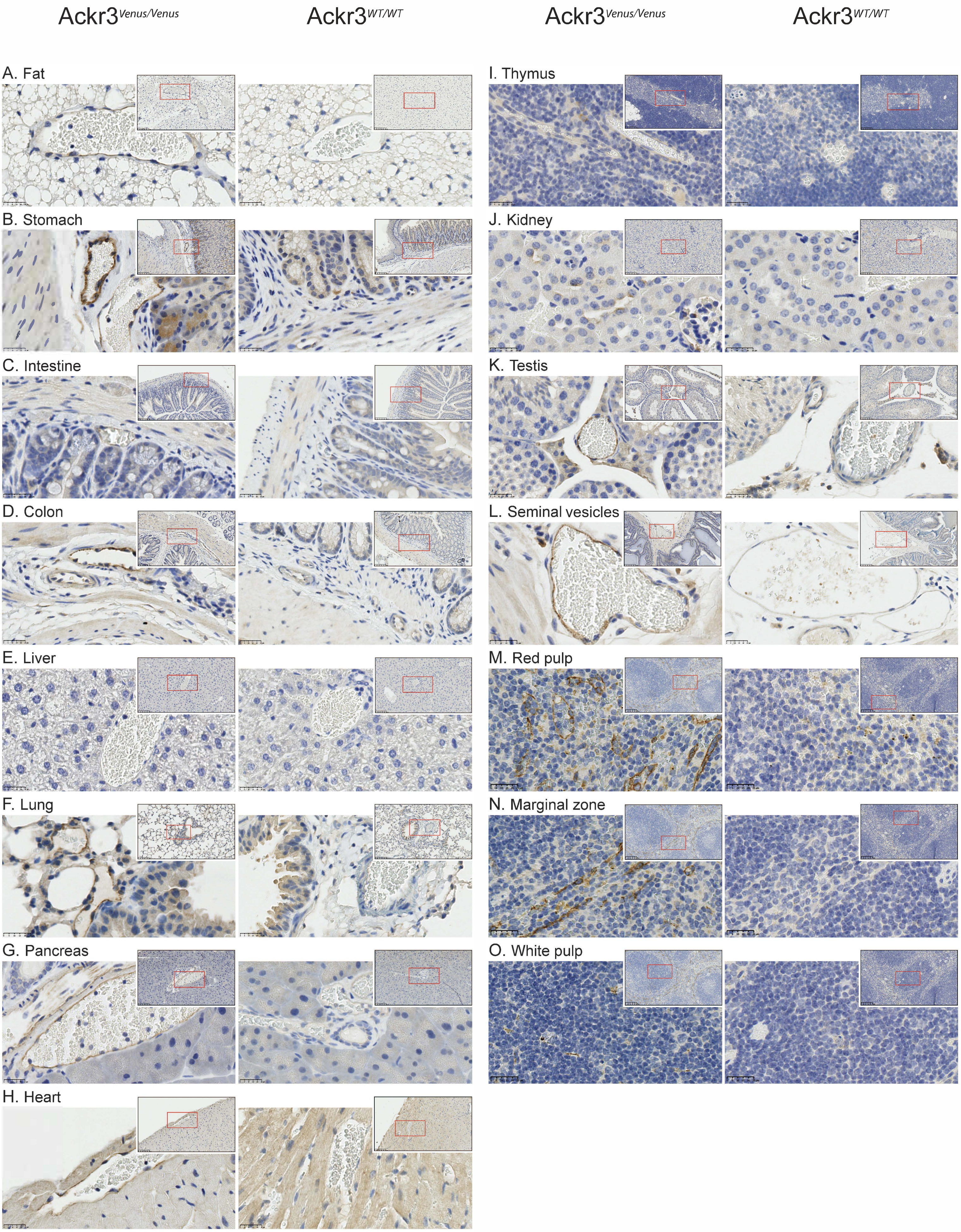

Supplemental Figure S6.

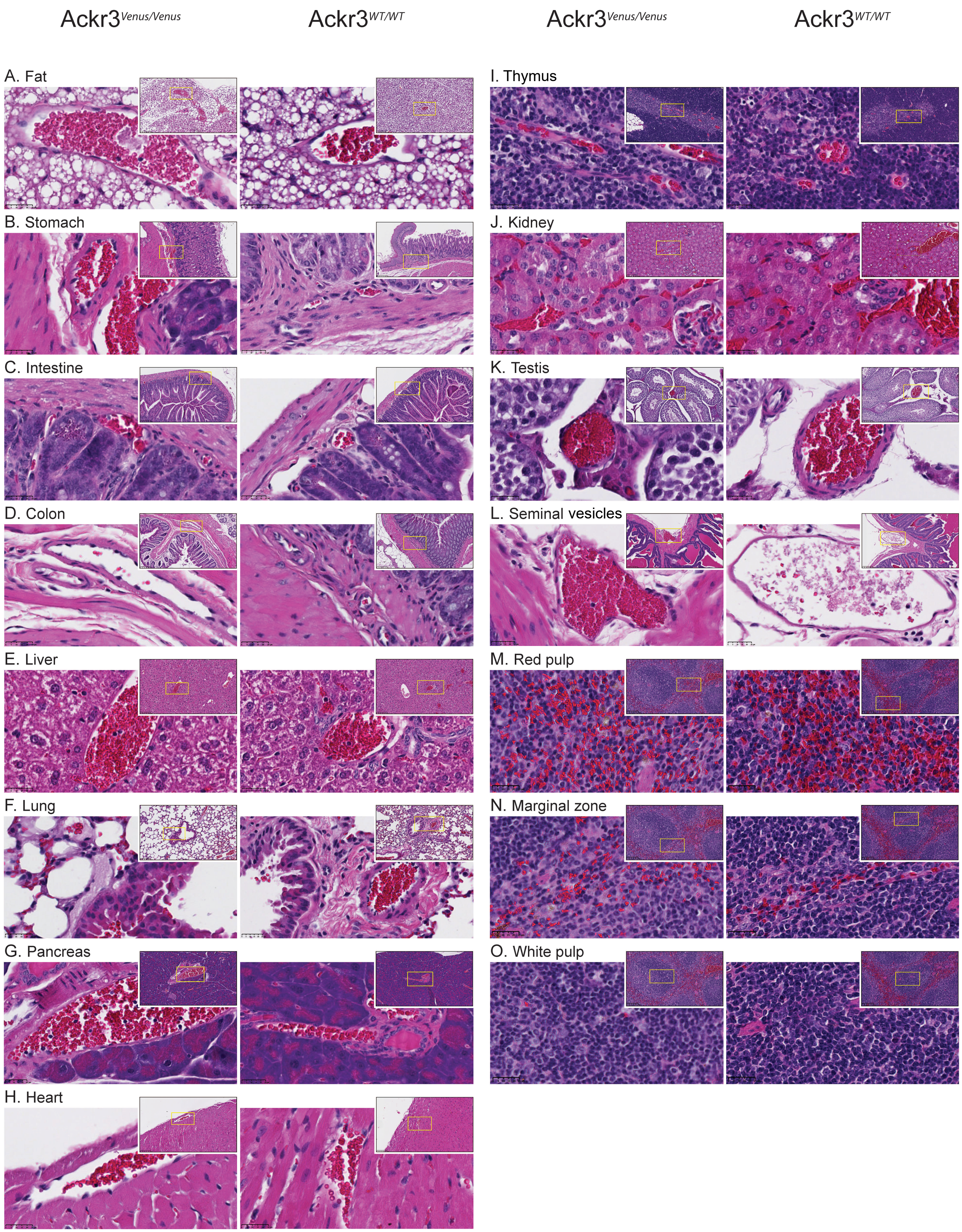

Supplemental Figure S7.

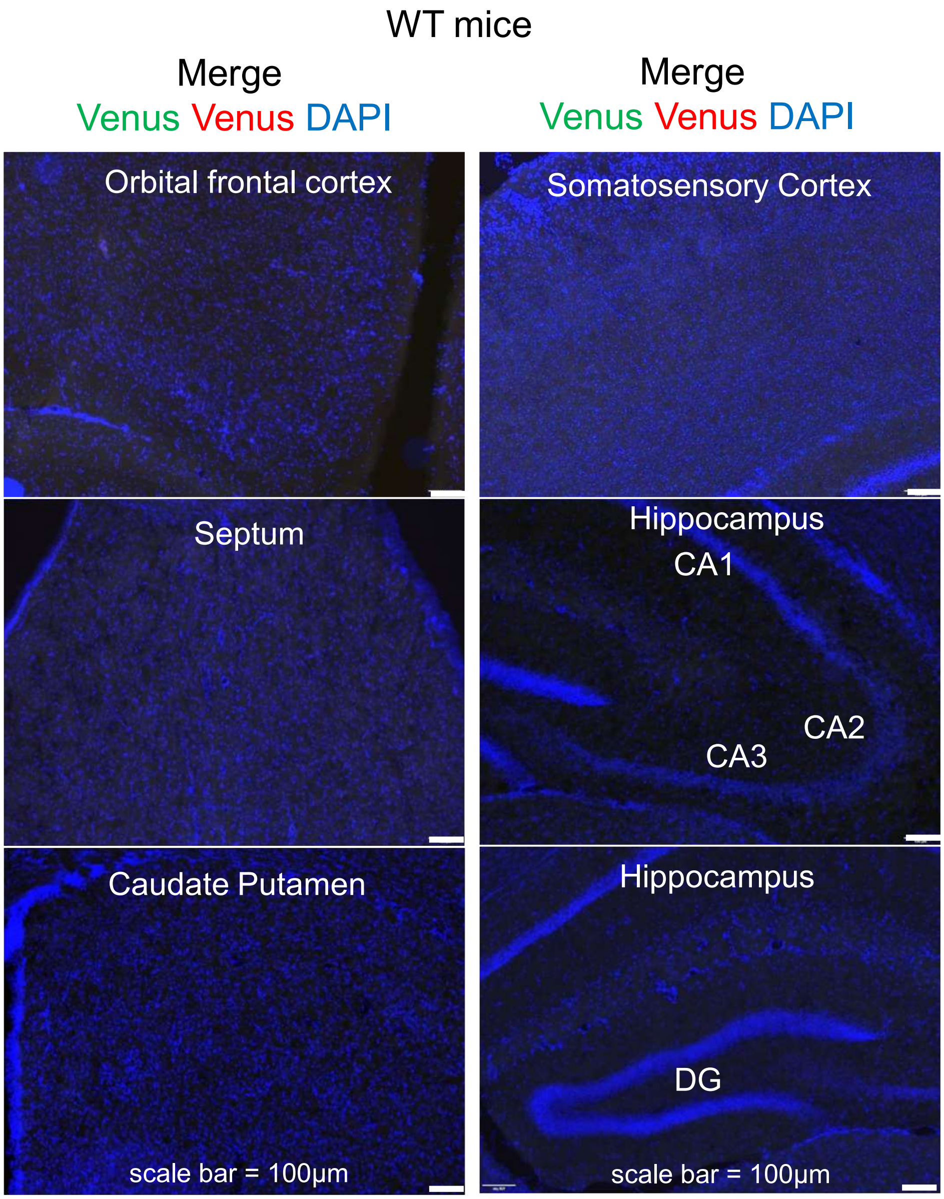

Supplemental Figure S8.

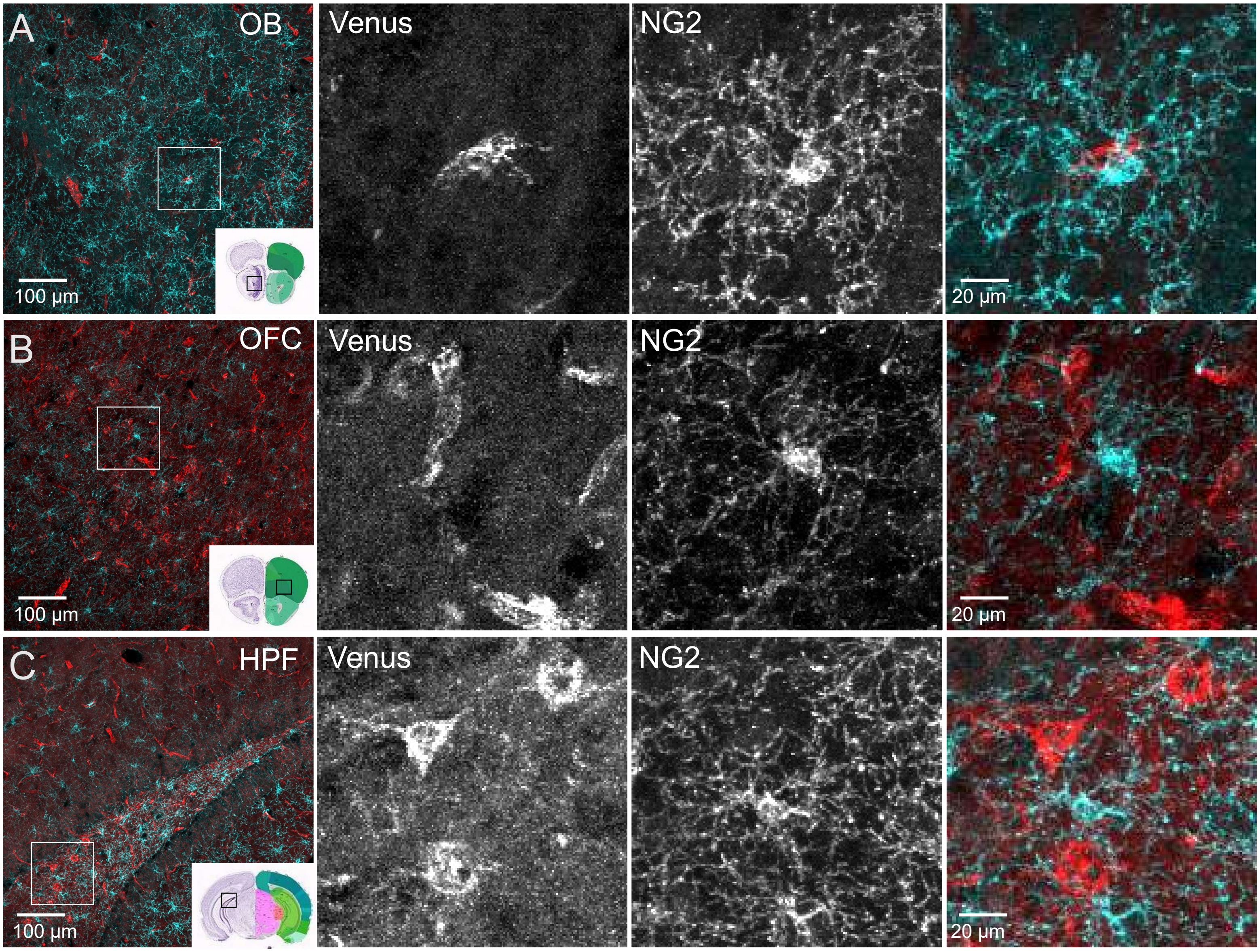

Supplemental Figure S9.
